# The circadian control of tryptophan metabolism regulates the host response to pulmonary fungal infections

**DOI:** 10.1093/pnasnexus/pgad036

**Published:** 2023-02-03

**Authors:** Claudia Stincardini, Marilena Pariano, Fiorella D’Onofrio, Giorgia Renga, Elena Orecchini, Ciriana Orabona, Emilia Nunzi, Marco Gargaro, Francesca Fallarino, Sung Kook Chun, Bridget M Fortin, Selma Masri, Stefano Brancorsini, Luigina Romani, Claudio Costantini, Marina Maria Bellet

**Affiliations:** Department of Medicine and Surgery, University of Perugia, P.le L. Severi 1, 06132 Perugia, Italy; Department of Medicine and Surgery, University of Perugia, P.le L. Severi 1, 06132 Perugia, Italy; Department of Medicine and Surgery, University of Perugia, P.le L. Severi 1, 06132 Perugia, Italy; Department of Medicine and Surgery, University of Perugia, P.le L. Severi 1, 06132 Perugia, Italy; Department of Medicine and Surgery, University of Perugia, P.le L. Severi 1, 06132 Perugia, Italy; Department of Medicine and Surgery, University of Perugia, P.le L. Severi 1, 06132 Perugia, Italy; Department of Medicine and Surgery, University of Perugia, P.le L. Severi 1, 06132 Perugia, Italy; Department of Medicine and Surgery, University of Perugia, P.le L. Severi 1, 06132 Perugia, Italy; Department of Medicine and Surgery, University of Perugia, P.le L. Severi 1, 06132 Perugia, Italy; Department of Biological Chemistry, University of California, Irvine (UCI), Irvine, CA 92697, USA; Department of Biological Chemistry, University of California, Irvine (UCI), Irvine, CA 92697, USA; Department of Biological Chemistry, University of California, Irvine (UCI), Irvine, CA 92697, USA; Department of Medicine and Surgery, University of Perugia, P.le L. Severi 1, 06132 Perugia, Italy; Department of Medicine and Surgery, University of Perugia, P.le L. Severi 1, 06132 Perugia, Italy; Department of Medicine and Surgery, University of Perugia, P.le L. Severi 1, 06132 Perugia, Italy; Department of Medicine and Surgery, University of Perugia, P.le L. Severi 1, 06132 Perugia, Italy

**Keywords:** circadian rhythm, IDO1, kynurenines, *Aspergillus fumigatus*, cystic fibrosis

## Abstract

The environmental light/dark cycle has left its mark on the body's physiological functions to condition not only our inner biology, but also the interaction with external cues. In this scenario, the circadian regulation of the immune response has emerged as a critical factor in defining the host–pathogen interaction and the identification of the underlying circuitry represents a prerequisite for the development of circadian-based therapeutic strategies. The possibility to track down the circadian regulation of the immune response to a metabolic pathway would represent a unique opportunity in this direction. Herein, we show that the metabolism of the essential amino acid tryptophan, involved in the regulation of fundamental processes in mammals, is regulated in a circadian manner in both murine and human cells and in mouse tissues. By resorting to a murine model of pulmonary infection with the opportunistic fungus *Aspergillus fumigatus*, we showed that the circadian oscillation in the lung of the tryptophan-degrading enzyme indoleamine 2,3-dioxygenase (IDO)1, generating the immunoregulatory kynurenine, resulted in diurnal changes in the immune response and the outcome of fungal infection. In addition, the circadian regulation of IDO1 drives such diurnal changes in a pre-clinical model of cystic fibrosis (CF), an autosomal recessive disease characterized by progressive lung function decline and recurrent infections, thus acquiring considerable clinical relevance. Our results demonstrate that the circadian rhythm at the intersection between metabolism and immune response underlies the diurnal changes in host–fungal interaction, thus paving the way for a circadian-based antimicrobial therapy.

**Significance Statement**

Circadian rhythm permeates all aspects of mammalian physiology, including the immune defense against infections. Here we report that the host response against *Aspergillus fumigatus* opportunistic fungal infection changes diurnally, an aspect particularly relevant in pathologies associated with aberrant immunity and inflammation, such as CF, characterized by increased predisposition to this type of infection. This diurnal change relies on the circadian regulation of the catabolism of the essential amino acid tryptophan driven by the enzyme IDO1. The circadian IDO1-mediated kynurenines production contributes to promote immune suppressive mechanisms at specific times of the day, thus modulating the antimicrobial host immune response.

## Introduction

The circadian clock is a highly conserved system that provides organisms with an internal molecular mechanism to anticipate environmental changes and adapt their physiology and metabolism to the external light–dark cycles. In humans and other mammals, a circadian pacemaker located in the suprachiasmatic nucleus of the brain maintains synchrony with clocks located in peripheral tissues. These cellular oscillators consist of transcriptional–translational feedback autoregulatory loops, in which transcriptional activators, CLOCK and BMAL1, induce the rhythmic expression of repressor genes Period (Per) and Cryptochrome (Cry), which form complexes to feedback and inhibit CLOCK/BMAL1-mediated transcription (1).

The circadian rhythm is increasingly being recognized as an important regulator of the immune response (2). Specific components of the circadian clock have been reported to control the expression and function of immune modulators and immune cells (3), and circadian regulation of host defense reactions against a variety of bacterial (4–6) and viral pathogens (7–10) and parasitic infections (11–13) has been demonstrated.

The metabolism of the essential aromatic amino acid tryptophan (Trp) plays a central role in the regulation of innate and adaptive immunity (14, 15). Three major pathways participate in Trp metabolism, leading to the production of kynurenine (Kyn), serotonin and indole derivatives (14). While the serotonin pathway is intimately linked to the circadian clock through its end-product melatonin, little is known about the interactions between the clock system and the Kyn pathway. This metabolic route accounts for 95% of Trp catabolism, the rate-limiting step being regulated by three enzymes, tryptophan 2,3-dioxygenase (TDO) and the two related indoleamine 2,3-dioxygenases (IDO1 and IDO2). While TDO and IDO2 are primarily expressed in the liver, IDO1 is detected throughout different peripheral tissues, including intestine, thymus, respiratory tract, spleen, placenta and different cells of the immune system (16). IDO1 in particular, has been the subject of intense research for its role in maintaining homeostasis and plasticity of the immune system, and its involvement in diseases ranging from allergy to infection and cancer (17). IDO1 immunoregulatory effects involve both Trp deprivation and Kyn production. On the one hand, Trp starvation suppresses acute inflammatory responses and promotes the generation of regulatory T cells (Treg); on the other hand, the product Kyn is a ligand for the Aryl hydrocarbon Receptor (AhR), a transcription factor of the bHLH-PAS family with a central role in immune functions (15).

In particular, the Kyn pathway has been involved in balancing resistance and tolerance to fungal infections (18) and decreased Trp catabolism, as a result of IDO1 deficiency, was causally linked to susceptibility to *Aspergillus fumigatus* infection in murine models of cystic fibrosis (CF), a monogenic autosomal recessive disorder in which the mutation in the CF transmembrane conductance regulator (CFTR) results in an exaggerated but ineffective airway inflammatory response that fails to eradicate pathogenic fungi (19, 20).

In the current study, we have demonstrated that IDO1 expression and activity are under circadian regulation in multiple tissues. In the lung, this time-dependent activation results in a significant diurnal change in the outcome of *A. fumigatus* infection. By resorting to a clinically relevant murine model of CF, we observed that, despite its reduced activity, *Ido1* maintains its circadian rhythmicity, thus driving a day–night response to infections in CF. These findings sustain a role of the circadian clock in controlling specific aspects of the host immune response to pathogens, a result that may guide novel therapeutic strategies in antimicrobial and anti-inflammatory treatments of patients.

## Results

### Tryptophan metabolism circadian oscillation in mouse tissues

**Fig. 1.**
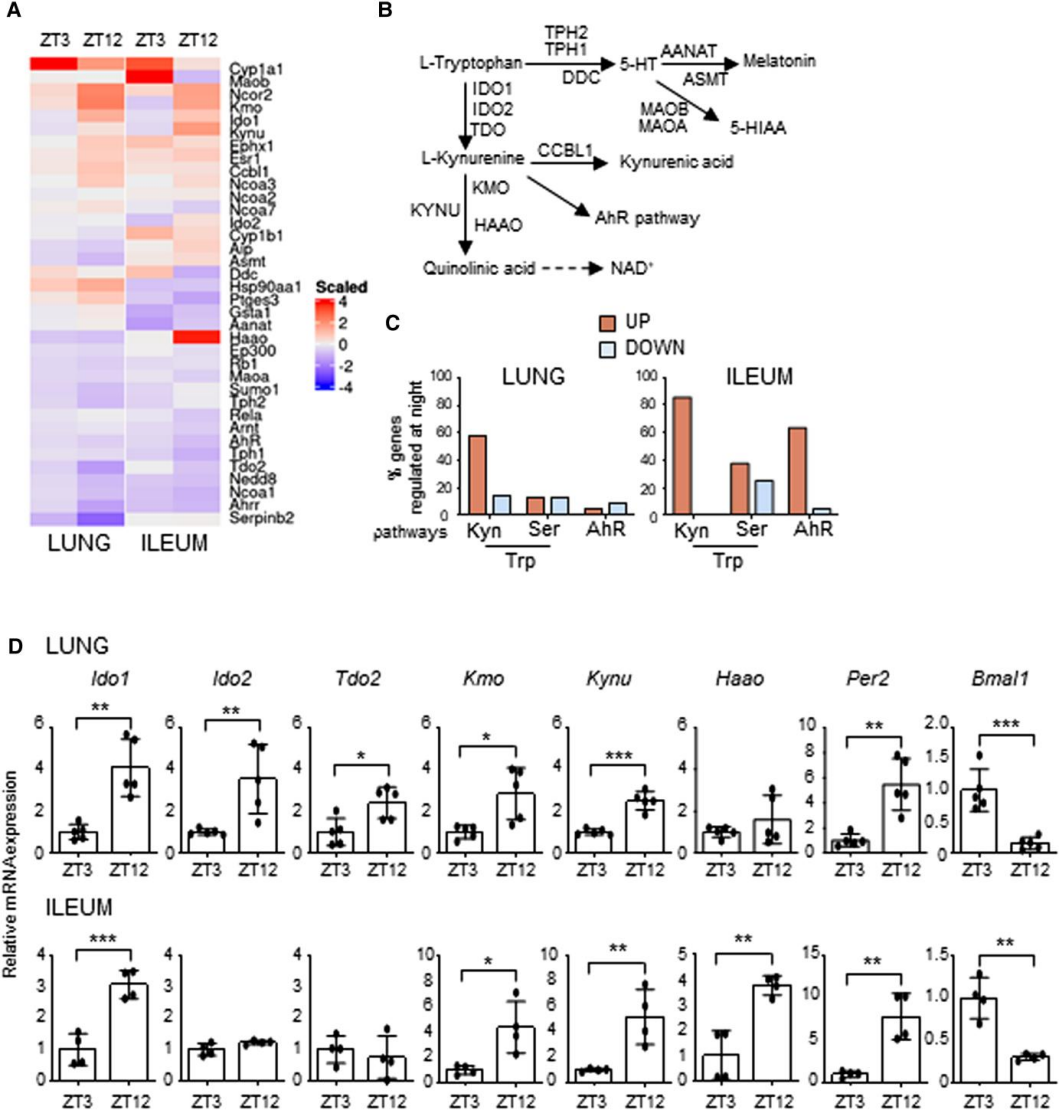
Time-of-day variation of the Trp metabolic pathway. (A) Heat diagram showing changes in gene expression detected by a custom QuantiGene plex gene expression assay in lungs and ilea of C57BL/6 wild-type mice collected at ZT3 and ZT12. Relative increase (red) or decrease (blue) of mRNA level is shown. Each time point includes three mice per group pooled before analysis. (B) Schematic representation of Trp metabolic pathways. (C) Diagram showing the % of genes whose basal levels are up- or down-regulated (>20%) at night versus day in the Trp-Kyn pathway, the Trp-Ser pathway or the downstream AhR pathway. (D) mRNA expression of *Ido1*, *Ido2*, *Tdo2*, *Kmo*, *Kynu*, *Haao*, *Per2,* and *Bmal1* in lung (n = 5) and ileum (n = 4) of C57BL/6 mice collected at ZT3 and ZT12. Data represent means ± SD. Student's *t*-test, significant changes are shown. *P < 0.05, **P < 0.01, ***P < 0.001.

**Fig. 2.**
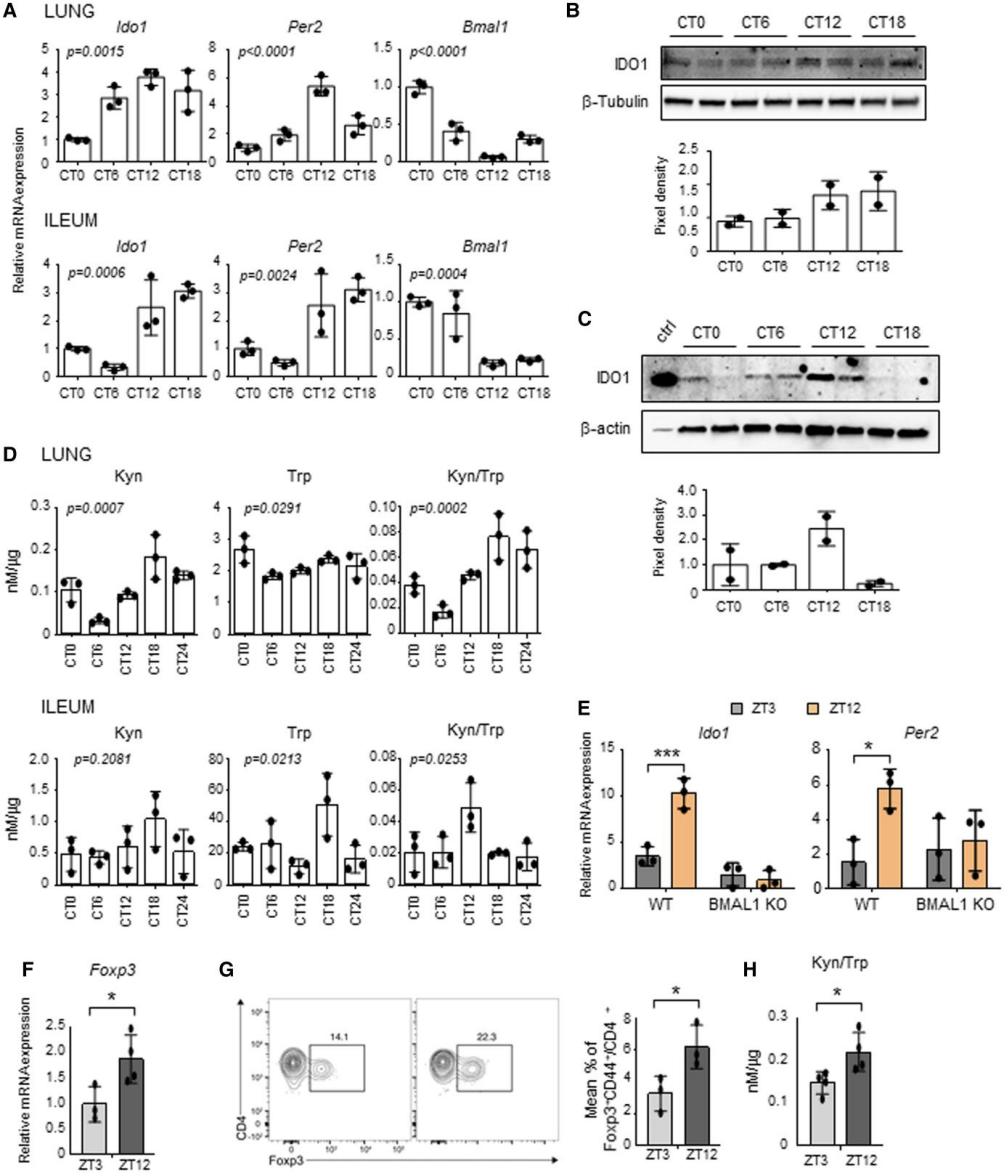
Circadian regulation of IDO1 expression and activity. (A-D) Lungs and ilea from C57BL/6 wild-type mice housed in constant darkness were collected at different circadian times (CT). A) *Ido1*, *Per2* and *Bmal1* expression profiles were analyzed by quantitative PCR. The values are relative to those of *beta-actin* mRNA levels at each CT. (B, C) Immunoblot and the relative densitometric analysis of IDO1 protein expression level was performed in lungs (B) and ilea (C). β-tubulin and β-actin were used as loading controls. Ctrl, IDO1 positive control. (D) Tissue homogenates were also assessed for kynurenine (Kyn) and tryptophan (Trp) levels, and Kyn/Trp ratio. All values are the mean ± SD (n = 3). One-way ANOVA, *P-*values are shown. E) *Ido1* and *Per2* gene expression were evaluated from ilea of villin-CRE conditional BMAL1 KO mice and isogenic wild-type taken at ZT3 and ZT12. The values are relative to those of 18S mRNA levels at each ZT. All values are the mean ± SD (n = 3). Two-way ANOVA, Bonferroni post-hoc test, significant changes are shown.*P < 0.05, ***P < 0.001. F-H) Quantification of *Foxp3* mRNA levels (F), Foxp3^+^ T cells by flow cytometry (G) and Kyn/Trp ratio (H) in lungs collected at the indicated ZTs. In (F) the values are relative to those of *beta-actin* mRNA levels at each ZT. In (G) the flow plot shows the % of Foxp3^+^ gated on CD4^+^ CD44^+^. Bar graph shows the % of total CD4+ T cells that express Foxp3. All values are the mean ± SD (n = 3–4). Student's t-test, significant changes are shown. *P < 0.05.

In order to determine how much of the Trp metabolism transcriptional pathway is regulated by the circadian system in peripheral tissues, we initially performed a QuantiGene Plex gene expression analysis in lung and ileum of mice housed in a 12-hour light/dark cycle and taken at zeitgeber time (ZT) 3 and ZT12 (Fig. 1A), where ZT0 is lights on and ZT12 is lights off (detailed description in the Materials and methods section). Between the transcripts analyzed (Fig. 1B), we observed that a larger percentage of the Kyn pathway, more than the serotonin pathway or the downstream AhR pathway, displayed day–night differences, with the majority of genes upregulated at ZT12 (Fig. 1C and Figures S1A and B). In particular, following qPCR validation, *Ido1*, *Ido2*, *Tdo2*, *Haao*, *Kmo,* and *Kynu* all displayed significant day–night difference in one or both tissues (Fig. 1D). IDO1, IDO2, and TDO in particular are rate-limiting enzymes of the Kyn pathway, converting Trp to Kyn. While IDO2 and TDO are constitutive enzymes more abundant in the liver, IDO1 is an inducible enzyme expressed in different tissues, including lung, intestine and cells of the immune system, where it plays a fundamental role in many physiological processes (16). For this reason, we focused on IDO1 to determine whether its transcriptional day–night change could reflect a circadian regulation of its gene. Mice were entrained on a 12-hour light: 12-hour dark cycle and then housed in constant darkness for 3 days before sacrifice. This revealed a clear circadian rhythmic change in *Ido1* expression in lung and ileum (Fig. 2A) as well as other tissues, including duodenum and spleen (Figure S1C). Interestingly, the enzymes *Tdo2* and *Ido2* also showed a significant 24-hour oscillation in the liver (Figure S1D). A time-dependent variation of *Ido1* mRNA expression was also observed in mouse embryonic fibroblasts (MEFs) in lung macrophages, and in human bronchial epithelial (HBE) cells synchronized in vitro by serum shock, as well as in cells obtained from naïve mice by broncoalveolar lavages (BALs) collected at two ZTs (Figure S2). *Ido1* mRNA circadian expression in lung and ileum resulted in the oscillation of IDO1 protein expression, albeit weak (Figs. 2B and C) and, more importantly, in a clear circadian oscillation in IDO1 activity, expressed by the conversion of Trp into Kyn, as detected by high-performance liquid chromatography (HPLC) in tissue homogenates (Fig. 2D). The oscillatory production of Kyn was also observed in the liver (Figure S1E). Since the enzymatic activity of IDO1 is correlated to its level of expression, we could speculate that the oscillation of Kyn production in lung and ileum, where IDO2 and TDO are poorly expressed, was mainly dependent on the oscillation of IDO1 expression. Importantly, the time-dependent *Ido1* mRNA variation was absent in ileum from mice with BMAL1 deletion in the gut (villin-CRE conditional BMAL1 KO mice) (21–23) (Fig. 2E), thus confirming that an intact intestinal local clock is required for the rhythmicity of *Ido1* gene expression. Both the increased Kyn production and Trp depletion induced by IDO1 activity control the conversion of naïve CD4+ T cells into Foxp3 + regulatory T (Treg) cells (24). Interestingly, both qPCR of *Foxp3* mRNA expression in lung tissues (Fig. 2F) and cytofluorimetric analysis of lung total cells collected at ZT3 and ZT12 (Fig. 2G) correlated with Kyn/Trp ratio diurnal change (Fig. 2H) and confirmed a significant difference in Foxp3+ Treg cells between the two time points.

We finally explored the possibility of a direct regulation of *Ido1* transcription by the circadian factors CLOCK and BMAL1, given the timing of E-box regulated genes (such as Per1/2) peaking at CT12–CT16. By searching for the presence of putative binding regions in the murine *Ido1* gene locus (−1 kb from the TSS), we found multiple non-canonical E-box response elements (Figure S3A). A reporter constituted by the *Ido1* gene promoter fused with the luciferase gene, was ectopically expressed in RAW 264.7 cells by transient transfection. However, co-expression of CLOCK, BMAL1, or CLOCK/BMAL1 did not result in the activation of the promoter (Figure S3B). A chromatin immunoprecipitation assay conducted in lungs taken at ZT3 and ZT12 confirmed that BMAL1 does not directly bind to those E-boxes (Figure S3C).

All together, these results confirmed that both the expression and activity of IDO1, one of the main enzymes involved in Trp catabolic pathway, are regulated in a circadian manner in multiple tissues. This time-dependent regulation likely contributes to immune homeostasis by generating an immune suppressive environment at specific circadian times.

### Diurnal modulation of pulmonary fungal colonization

**Fig. 3.**
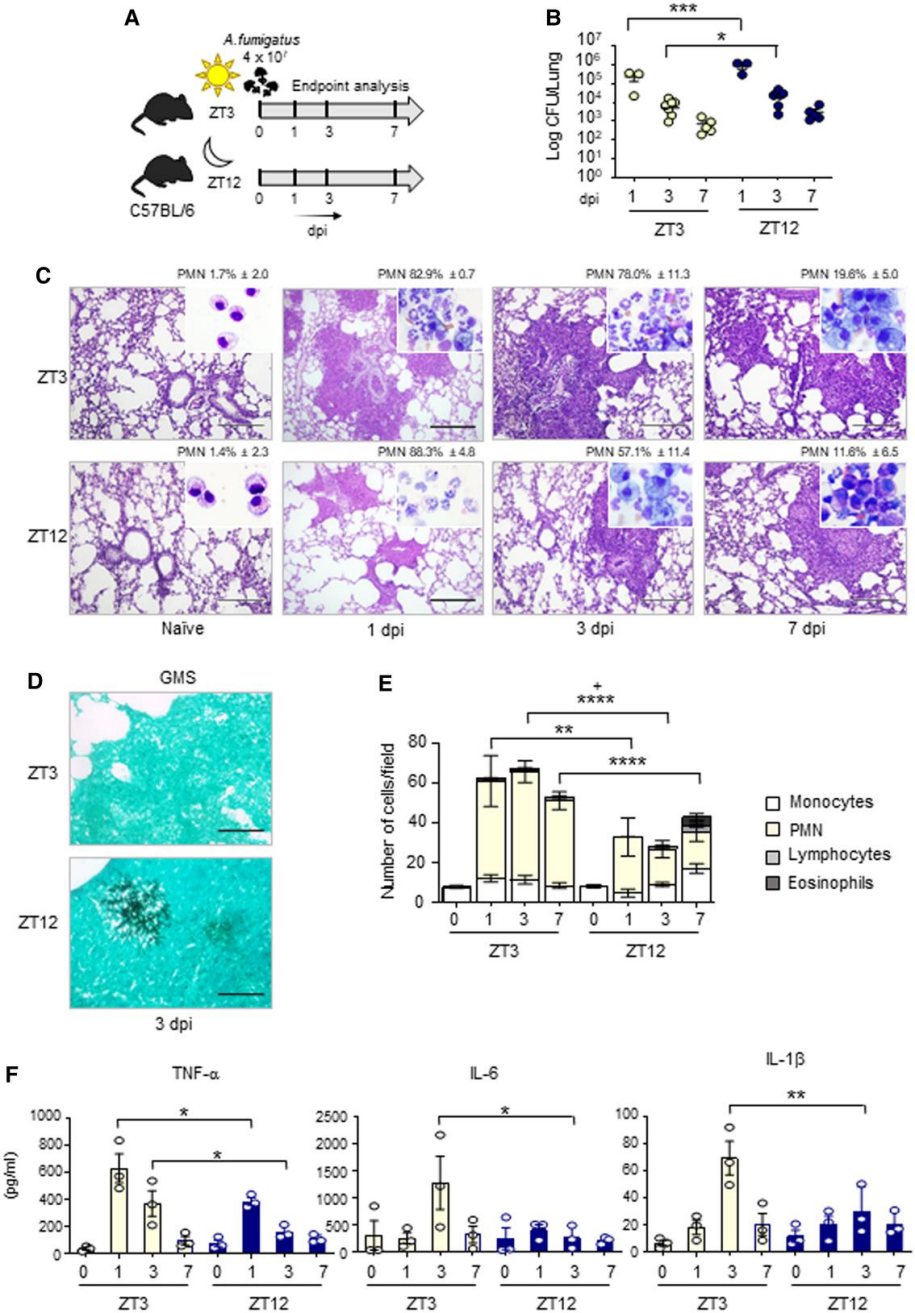
Diurnal regulation of the host response to *A. fumigatus* infection. C57BL/6 wild-type mice (n = 3–7 mice per group from two independent experiments) were infected intranasally with live *A. fumigatus* conidia at two ZT, and assessed at 1, 3, and 7 day post-infection (dpi), as described in the experimental plan (A). (B) Lung fungal growth studied by the count of colony forming units (log10 CFU). Black bars indicate the geometric mean (n = 3–7). *P*-values were generated by two-way ANOVA test, Bonferroni post-hoc test, significant changes are shown. *P < 0.05, ***P < 0.001. (C) Lung histology studied by periodic acid–Schiff staining (left, scale bars 200 μm), and percentage of polymorphonuclear neutrophils studied by May–Grünwald Giemsa staining of cells obtained from BAL (insets). (D) Lung fungal infiltration at 3 dpi studied by Grocott-Gomori methenamine staining (right, scale bars 100 μm). (E) BAL cellular morphometry, expressed as means of total and differential cell counts ± SEM are presented. *P*-values were generated by two-way ANOVA test, Bonferroni post-hoc test, significant changes are shown. **P < 0.01, ****P < 0.0001 for PMN, ^+^*P* < 0.05 for total cells. (E) Mouse pro-inflammatory cytokines measured by multiplex immunoassay in lung homogenates. Results shown as mean ± SEM (n = 3). *P*-values were generated by two-way ANOVA test, Bonferroni post-hoc test. Significant changes are shown. *P < 0.05, **P < 0.01.

The balance between tolerance and inflammation in fungal infection is strictly controlled by the metabolic pathway involved in Trp catabolism mediated by IDO1 (18). For this reason, in order to define the importance of *Ido1* circadian oscillation in a physio-pathological context, we resorted to a model of lung infection with the opportunistic fungus *A. fumigatus*. We intranasally infected mice at ZT3 and ZT12, time of low and high expression of *Ido1*, and collected tissues at 1, 3, and 7 days post-infection (sacrifice of mice infected at ZT3 was performed at ZT3; sacrifice of mice infected at ZT12 was performed at ZT12) (Fig. 3A). Time-of-day dependent differences in fungal colonization were examined in lungs, as well as brain, an organ where fungal dissemination frequently occurs. We obtained data showing that lung colonization was significantly higher at night than during the day at 1 and 3 days post-infection (dpi), as revealed by the fungal outgrowth from lung homogenate (Fig. 3B), and some persistence of brain dissemination at 7 dpi (Figure S4A). Lung sections from mice infected with *A. fumigatus* at ZT3 showed lesions characterized by infiltration of immune cells and disruption of the alveolar architecture. Instead, infection at ZT12 was associated with a moderate infiltration with immune cells (Fig. 3C). However, in accordance with CFU count, an increased number of *A. fumigatus* hyphae was detectable in lung sections from mice infected and collected at ZT12 through Grocott-Gomori stain (Fig. 3D). Cytospin preparation from cells obtained by BALs revealed an immune cell infiltrate with increased neutrophil recruitment at daytime compared to mice infected at ZT12 (Figs. 3C and E). qPCR and Multiplex immunoassay performed in lung homogenates confirmed that, in line with cytospin analysis, infection at ZT12 was associated with lower levels of expression of pro-inflammatory cytokines TNFα, IL-6, and IL-1β (Fig. 3F) and the chemokine CXCL1 (Figures S4B and S5). Interestingly, we observed a large increase of eosinophils in mice infected at ZT12 at 7 dpi (Fig. 3E). This correlated with increased levels of the eosinophil chemotactic protein eotaxin at the same time point (Figure S4B).

Previous studies have described how the circadian clock in macrophages controls pro-inflammatory cytokines secretion in response to different stimuli (25, 26). However, in lung macrophages synchronized in vitro by serum shock and pulsed with *Aspergillus* or with the fungal component zymosan, cytokines secretion was not different between the two time points analyzed (Figure S6), suggesting that the intrinsic macrophage rhythm was not involved in the circadian pulmonary response to *A. fumigatus*.

Recognition of fungi by the innate immune system is mainly mediated by Toll-like receptors, Nod-like receptors and C-type lectin receptors. Dectin1 in particular is the main Pattern Recognition Receptor that recognizes β-glucans in fungal cell wall and, following ligation, it induces the production of pro-and anti-inflammatory cytokines and chemokines. However, as previously demonstrated (27), no time-dependent variations in the basal levels of *Dectin 1*, *Tlr2, Tlr4, Nlrp3,* and *Cd11b* gene expression were observed in lung tissues (Figure S5) or in macrophages (Figure S6). This result suggests that the day–night difference in fungal colonization was unlikely to involve a rhythmic regulation of fungal PRRs expression.

To further corroborate our findings, we also used a second model of fungal gastrointestinal infection. Similarly to what observed in the lung, intragastric infection of *Candida Albicans* performed at ZT3 and ZT12 in wild-type mice suggested increased colonization at ZT12 at 3 and 10 dpi (Figure S7), a result that, if confirmed with a higher group number, would reveal a possible common circadian mechanism of control of fungal growth.

**Fig. 4.**
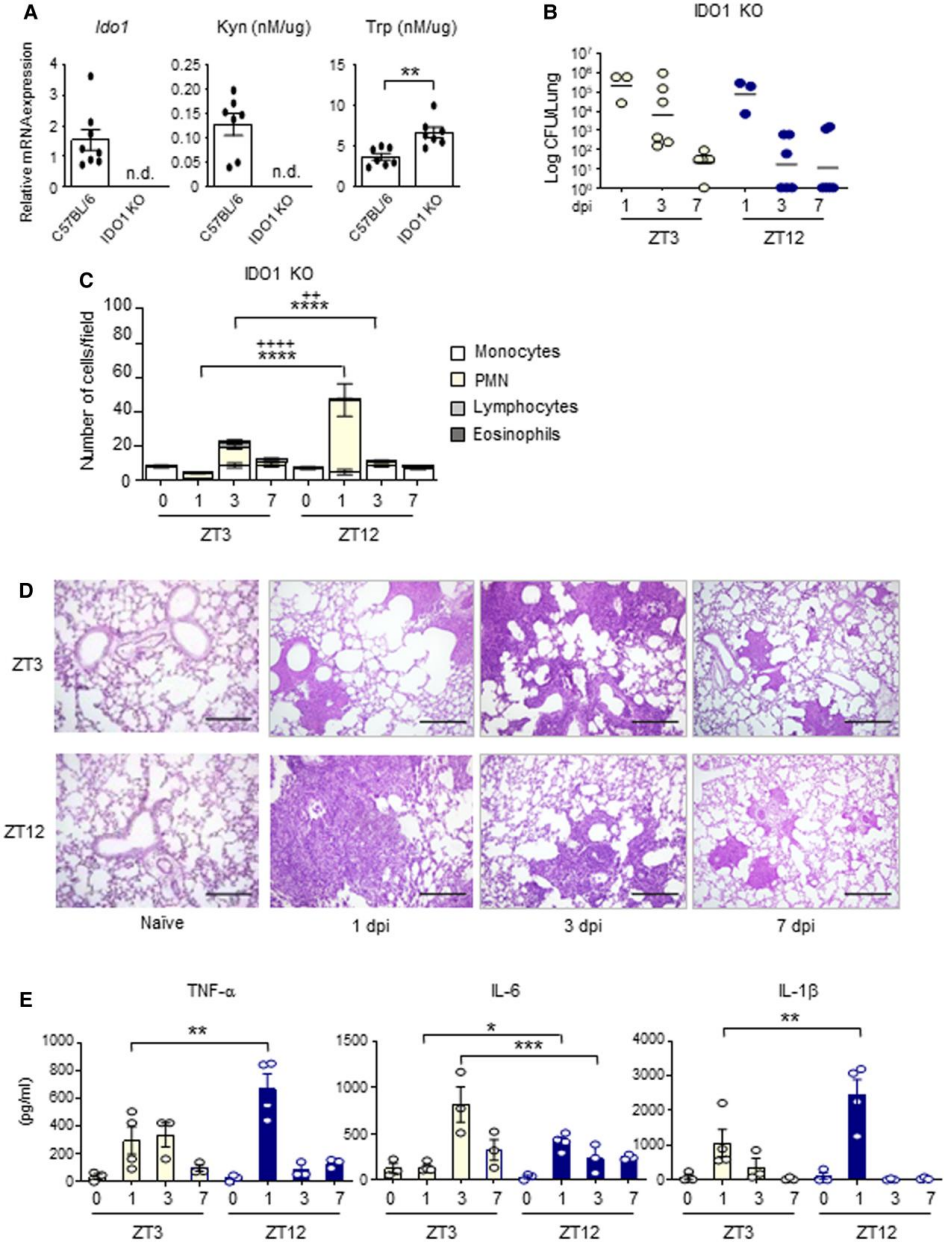
IDO1-dependent day–night changes of the host response to *A. fumigatus* infection. (A) *Ido1* gene expression, Kyn, and Trp levels in lung homogenates from WT and IDO1 KO mice. All values are the mean + SEM (n = 7–8). Student's *t*-test, significant changes are shown. **P < 0.01, n.d., not detectable. (B-E) IDO1 KO mice (n = 3–6 mice per group) were infected intranasally with live *A. fumigatus* conidia at two ZT, together with C57BL/6 wild-type described in Fig. 3. (B) Lung CFU were assessed at 1, 3, and 7 dpi in IDO1 KO. Black bars indicate the geometric mean (n = 3–6). *P*-values were generated by two-way ANOVA test, Bonferroni post-hoc test. No statistically significant changes were found. (C) BAL cellular morphometry, expressed as means of total and differential cell counts ± SEM are presented. *P*-values were generated by two-way ANOVA test, Bonferroni post-hoc test, significant changes are shown. ****P < 0.0001 for PMN, ++++ *P* < 0.0001 and ++ *P* < 0.01 for total cells. (D) Lung histology studied by periodic acid–Schiff staining. Scale bars 200 μm. In (E), mouse pro-inflammatory cytokines measured by multiplex immunoassay in lung homogenates of IDO1 KO mice. (n = 3–4). All the values are the mean ± SEM, *P < 0.05, **P < 0.01, ***P < 0.001. Two-way ANOVA, Bonferroni post-hoc test.

In order to define the role of IDO1 in the observed day–night differences, we investigated whether fungal proliferation in lungs was affected in the absence of IDO1. Indeed, IDO1 KO mice showed negligible levels of Kyn in the lungs while Trp consumption was reduced compared to wild-type mice (Fig. 4A), confirming the importance of IDO1 enzymatic activity in this organ. By infecting IDO1 KO mice with *A. fumigatus* at ZT3 and ZT12, we could not observe significant diurnal differences in terms of lung fungal colonization at 1, 3, and 7 dpi compared to WT control mice infected at the same time (Fig. 3). Indeed, while in WT mice fungal growth was significantly greater at night since early infection times, IDO1 KO mice infected at ZT12 always displayed low CFU count (Fig. 4B) and increased resistance to lung colonization, as shown by the higher PMN recruitment (Fig. 4C), lung inflammatory cells infiltration (Fig. 4D) and cytokines expression (Fig. 4E) observed early 1 day following infection. All together, these results suggested that the host response to lung infection with A*. fumigatus* follows a diurnal pattern of activation, to which IDO1 circadian activity could contribute by reducing the magnitude of the inflammatory response when IDO1 is more active. Indeed, in mice infected at ZT12, a reduced fungal clearance was associated with a defective fungal-load dependent inflammatory response, possibly driving an evolution towards allergy.

### The time-dependent induction of IDO1 modulates the outcome to *Aspergillus fumigatus* infection

**Fig. 5.**
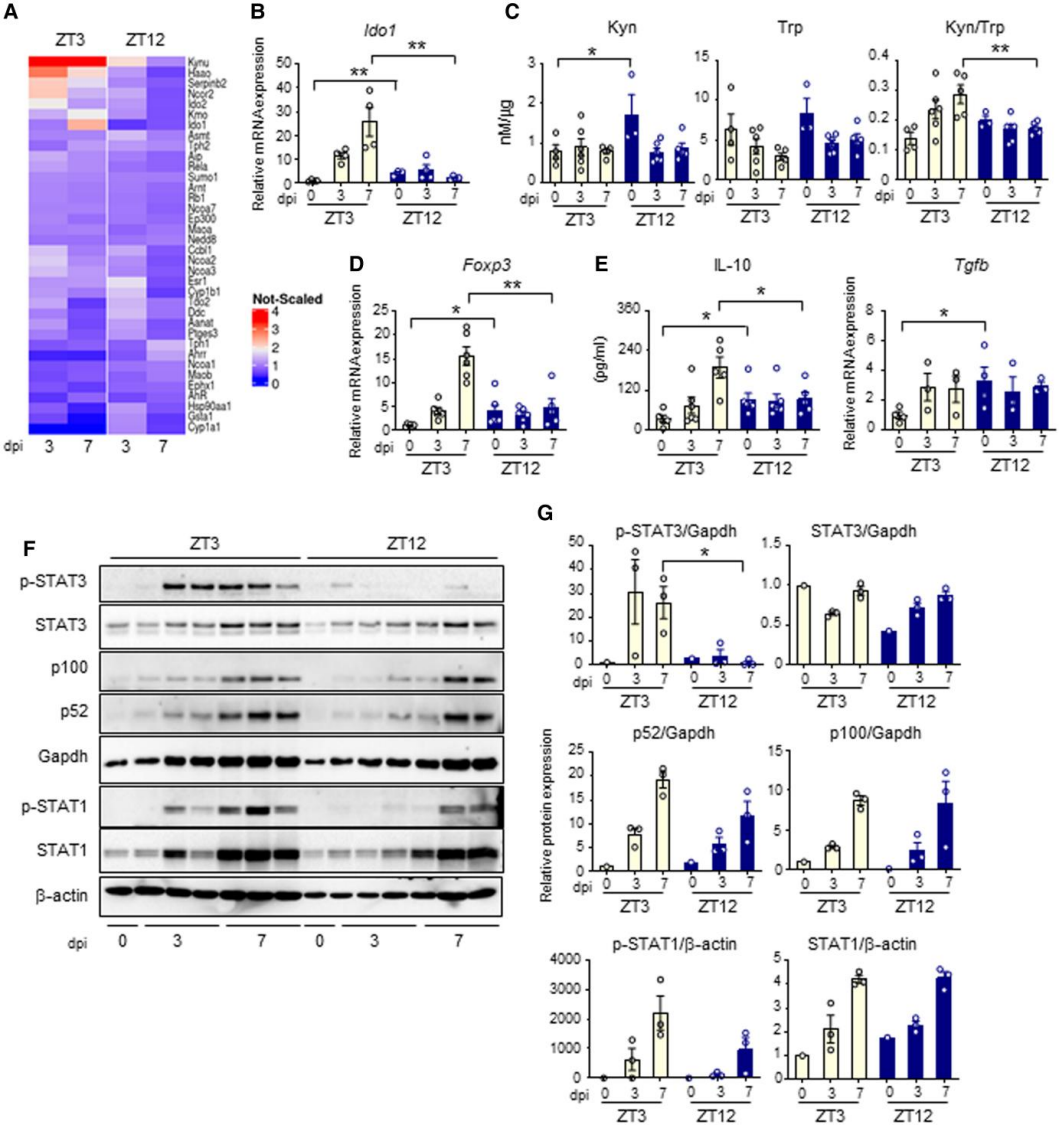
Day–night induction of the IDO1-kyn pathway during *A. fumigatus* infection. (A) Heat diagram showing changes in gene expression detected by a custom QuantiGene plex gene expression assay in lungs of C57BL/6 WT mice infected at ZT3 and ZT12 as in the experimental plan of Fig. 3A. Relative increase (red) or decrease (blue) of mRNA level is shown. (B-E) Evaluation of *Ido1* (B), Kyn, Trp and Kyn/Trp ratio (C), *Foxp3* (D), IL-10 and *Tgfb* production (E) in lung homogenates from C57BL/6 mice collected at the indicated time points. All the values are the mean ± SEM (n = 3–8), *P < 0.05, **P < 0.01. Two-way ANOVA, Bonferroni post-hoc test and Student's *t*-test. (F-G) Evaluation of JAK-STAT pathway (p-STAT3, STAT3, p-STAT1, STAT1) and non-canonical NF-kB pathway (p100/p52) through immunoblotting in lung homogenates. Diagrams on the right show densitometric analysis. All the values are the mean ± SEM (n = 3), Student's *t*-test, *P < 0.05.

To better elucidate how IDO1 circadian regulation influences the host response to *Aspergillus* infection, we analyzed the transcriptional induction of the Trp metabolic pathway during infection. QuantiGene Plex gene expression analysis clearly revealed induction of *Ido1* and other genes of the Kyn pathway (*Kynu*, *Kmo*, *Haao*) in mice infected at ZT3, and only a weak induction or no induction at ZT12 (Fig. 5A). qPCR analysis confirmed that *Ido1* mRNA expression was strongly induced during daytime infection, with a peak at 7 dpi (Fig. 5B). At ZT12, despite the high basal level, *Ido1* was not further induced and remains constant during infection. In accordance with this, Trp consumption in the lung was increased at ZT3, but not ZT12, thus resulting in increased Kyn/Trp ratio and increased IDO1 activity at the same time point (Fig. 5C).

An increased IDO1 activity usually results in an immunosuppressive phenotype through, among all, the inhibition of Th cells and the generation of regulatory T cells, one function of which is to restrain pathological inflammation. While we did not measure significant differences in Th cells activation, obtained by measuring levels of Th1, Th2, and Th17 cytokines in lungs homogenates (Figure S8), we observed increased levels of Treg marker *Foxp3* (Fig. 5D) and increased suppressive cytokine IL-10, (Fig. 5E) at 7 dpi in mice infected at ZT3 compared to those infected at ZT12. Instead, the expression of another suppressive cytokine, *Tgfb*, was higher at ZT12 in uninfected mice, and was not further modulated during infection (Fig. 5E). To investigate the mechanism involved in the diurnal induction of IDO1, we evaluated the level of activation of the JAK-STAT pathway and the non-canonical NF-κB pathway, which are known to induce IDO1 during inflammatory conditions (28). We observed that STAT3 phosphorylation, more than STAT1 phosphorylation or p100 cleavage to p52, was significantly higher in mice infected at ZT3 than at ZT12, suggesting a potential role of STAT3 in IDO1 time-dependent induction (Figs. 5F and G). All together, these results revealed a diurnal regulation of the IDO1-Kyn pathway during lung infection with *A. fumigatus.* In mice infected at ZT3, when inflammatory STAT-dependent pathways are more strongly activated, IDO1 was induced more efficiently and promoted a late immunosuppressive response. At ZT12, when high basal level of IDO1 restrain the starting of the inflammatory response, IDO1 was not efficiently induced beyond its basal expression.

### The circadian regulation of IDO1 influences the outcome of *Aspergillus fumigatus* infection in CF

In order to establish the potential relevance of our findings in a clinical condition characterized by increased susceptibility to pulmonary infections, including fungal infections, we resorted to a murine model of CF, harboring the most frequent *Cftr* mutation F508del (*Cftr^F508del^* mice or CF mice). In mice with CF, a defective IDO1 activity in the lung (Figure S9A) has already been demonstrated as relevant in influencing the outcome of lung infections (29) because its reduced activity favors a Th17/Treg imbalance that is causally linked to a pathogenic non-protective response to the fungus.

**Fig. 6.**
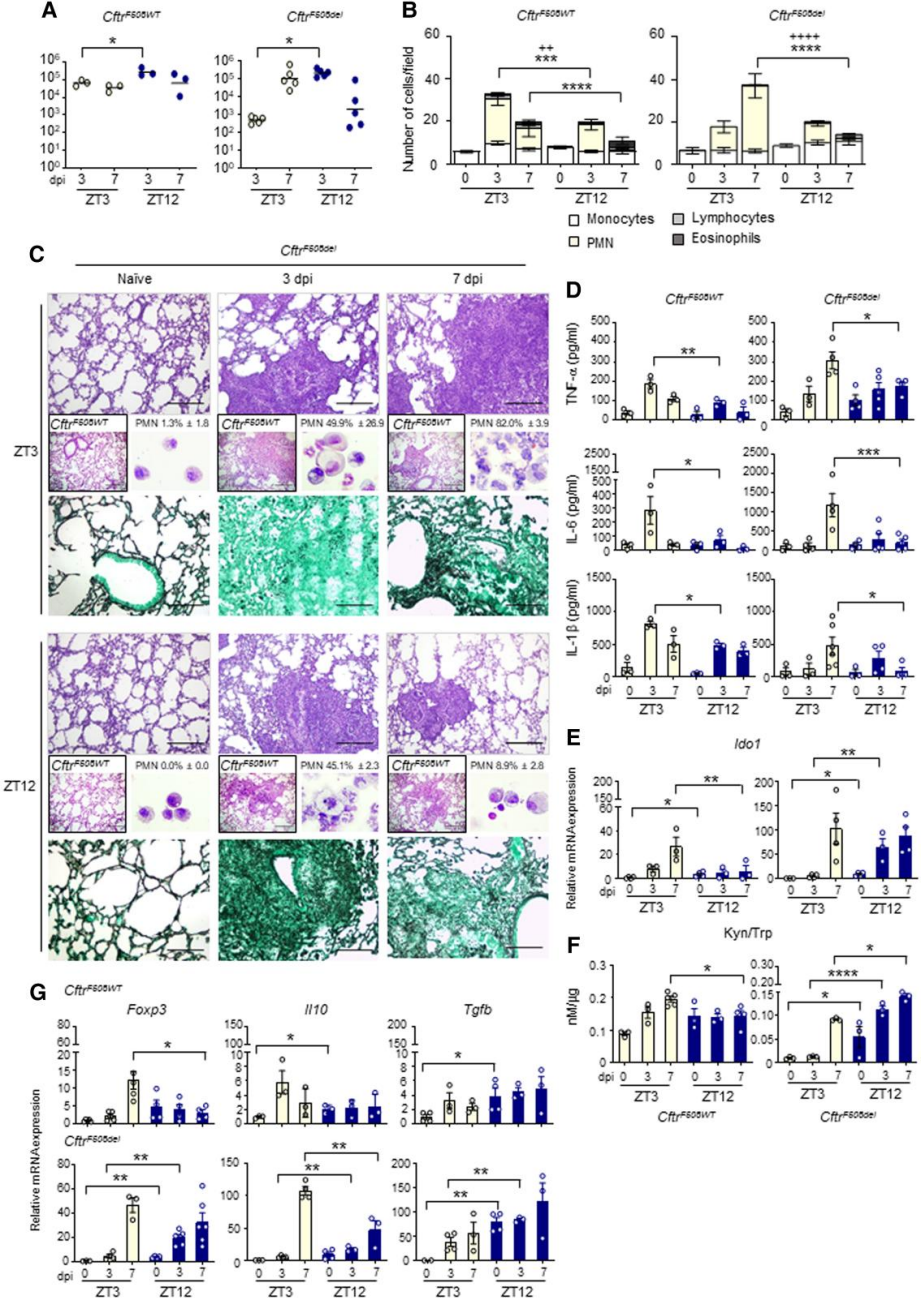
Circadian IDO1 modulates the response to *A. fumigatus* infection in CF mice. Homozygous F508del-Cftr C57BL/6 mice (referred to as *Cftr^F508del^* mice) and isogenic WT (*Cftr^WT^*) were infected at ZT3 and ZT12 and assessed at 3 and 7 dpi. (A) Fungal growth in the lung (log10 CFU). Black bars indicate the geometric mean (n = 3–5). *P*-values were generated by two-way ANOVA test, Bonferroni post-hoc test, significant changes are shown. *P < 0.05. (B) BAL cellular morphometry expressed as means of total and differential cell counts ± SEM are presented. *P*-values were generated by two-way ANOVA test, Bonferroni post-hoc test, significant changes are shown. ***P < 0.001, ****P < 0.0001 for PMN, ^++^*P* < 0.01, ^++++^*P* < 0.0001 for total cells. (C) Lung histology studied by periodic acid–Schiff staining in *Cftr^WT^* (left boxed insets) and *Cftr^F508del^* mice (scale bars 200 μm); lung fungal infiltration studied by Grocott-Gomori's methenamine silver stain (scale bars 100 μm) and percentage of polymorphonuclear neutrophils studied by May–Grünwald Giemsa staining of cells obtained from BAL (right insets) in *Cftr^F508del^* mice. (D) Mouse pro-inflammatory cytokines measured by multiplex immunoassay in lung homogenates. Results shown as mean ± SEM (n = 3–6). *P*-values were generated by two-way ANOVA test, Bonferroni post-hoc test. Significant changes are shown. *P < 0.05, **P < 0.01, ***P < 0.001. (E-G) *Ido1* mRNA expression (E) and activity (F), *Foxp3*, *Il10* and *Tgfb* gene expression in lung homogenates (G). The values are the mean ± SEM (n = 3–6). Two-way ANOVA, Bonferroni post-hoc test and Student's t-test, significant changes are shown, *P < 0.05, **P < 0.01, ***P < 0.001.

In *Cftr^WT^* mice, similar to what observed in Fig. 3, lung colonization at 3 dpi was slightly but significantly higher at ZT12 compared to ZT3 (Fig. 6A). In lungs of *Cftr^F508del^* mice infected at ZT3, a progressive increase in CFU number was observed (Fig. 6A) and was associated with a sustained and persistent inflammatory response characterized by high neutrophil recruitment in lung and the BAL (Figs. 6B and C), high hyphal infiltration of the lung (Fig. 6C) and high levels of pro-inflammatory cytokines TNF-α, IL-6, and IL-1β at 7 dpi (Fig. 6D). Nonetheless, the time-dependent differences in fungal colonization between ZT3 and ZT12 were confirmed in CF lungs (Fig. 6A), as well as a significantly different level of inflammatory cell recruitment and inflammatory cytokines secretion, all reduced at night time at 7 dpi (Fig. 6B-D). Interestingly, we observed that *Ido1* transcription remained circadian in CF mice (Figure S9B), as well as Kyn production (Figure S9C) and Kyn/Trp ratio, although to reduced levels (Figure S9D), with no apparent variations in period and amplitude compared to wild-type mice. The expression and activity of IDO1 during infection were maintained stably greater at night than during the day in CF mice (Figs. 6E and F), as well as levels of *Foxp3*, *Il10* and *Tgfb* gene expression (Fig. 6G), which may explain the reduced inflammatory damage and PMN recruitment in mice infected at ZT12. Taken together, these results pointed to an important role played by the time-dependent immunity and tolerance to *Aspergillus* in CF.

## Discussion

Although the relevance of Trp catabolic pathways in both the regulation of immune functions against infections and in sleep and circadian physiology is widely accepted, a direct connection between the circadian system and the Kyn pathway in peripheral tissues has remained elusive. An oscillatory expression of some Kyn pathway enzymes has been documented in rat pineal gland (30). Furthermore, a very recent work has demonstrated how Trp metabolites regulate physiological circadian rhythms in both the liver and the SCN (31). Here we demonstrated that IDO1, the main rate-limiting enzyme of the Kyn pathway, is expressed in a circadian manner in multiple peripheral tissues in both healthy mice and fungal infected mice, and we showed the relevance of this oscillation in the modulation of the antimicrobial immune response.

First, the circadian regulation of basal IDO1 levels may suggest the presence of more active surveillance mechanisms at the times of day when the encounter with microorganisms is most likely. Lungs are daily exposed to a variety of particles, allergens, and airborne microbes. Airway tolerance is a state of immunological surveillance that prevents the unwanted development of lung inflammation and allergic diseases after inhalation of harmless environmental antigens. Evidences suggest that Treg cells expressing the transcription factor Foxp3 are key elements for maintaining this suppressive environment in the lung, and the importance of IDO1 activation in Treg generation is widely recognized. Here we propose that the oscillatory expression and activity of IDO1 at the steady state condition, with a peak of expression at the beginning of the active phase in mice, may have developed to help maintain this state of airway (and gastrointestinal) tolerance at specific time of the day, when the possibility to encounter multiple microorganisms is increased. *Aspergillus* species contaminate the environment but they rarely infect mammals. Many different environmental and physiological conditions can contribute to the development of fungal disease, and circadian variation of the host immune status is likely to be one of them. In particular, our results suggest a role for IDO1 circadian regulation in the definition of the time-dependent susceptibility of mice to *A. fumigatus* infection. We observed that high levels of IDO1 at the beginning of the dark phase contributes to the increase of Tregs in naïve tissues at the same time point, thus influencing the capability of colonization of opportunistic pathogens such as *Aspergillus*.

Secondly, the time-dependent activation of the Kyn pathway during fungal infection fits into a well-known broader circadian regulation of immune functions. Here, we showed that the host response to *Aspergillus* infection profoundly change its magnitude and efficiency depending on the time of first contact with the microorganism. This differential response, as observed in a variety of studies using many different models of infections, is driven by the circadian clock controlling many aspects of the immune response, and we propose that the regulation of IDO1 expression is one of them. IDO1 is expressed in a variety of immune cells, including dendritic cells and monocyte-derived cells, and is induced during infection by several pro-inflammatory cytokines through IFN-γ-dependent or independent mechanisms. Here we observed that a STAT-driven pro-inflammatory signaling pathway may be involved in the differential induction of IDO1 at different time of the day.

However, the transcriptional mechanism by which the circadian clock regulates *Ido1* gene expression remains to be determined. Our results did not confirm the presence of a direct regulation of *Ido1* expression by CLOCK/BMAL1 and seems to point towards an indirect circadian regulation involving transcription factors other than CLOCK/BMAL1, such as IRF1, STAT1, or RelB, which are all involved in *Ido1* transcriptional regulation (32). RelB in particular might be an ideal candidate, as it is known to modulate *Ido1* expression in dendritic cells together with AhR (33) and we previously reported that it physically interacts with BMAL1 and participates in the modulation of circadian gene expression (34). However, considering the full length of murine *Ido1* promoter spanning from over 5000 bp upstream to more than 2000 bp downstream to *Ido1* start codon, we cannot exclude that CLOCK/BMAL1 may bind regions of the *Ido1* promoter not yet analyzed. Considering that the activity of IDO1 is able to modify the inflammatory state during infectious diseases, understanding in detail the mechanisms of its circadian regulation will be of great interest for the design of therapeutic strategies in different immune-related pathologies.

Together with Trp metabolism, another amino acid whose metabolism is associated with important immunoregulatory effects is arginine (Arg) (35). IDO1 and Arg metabolic pathways often interacts while playing their specific immunoregulatory roles. First, they can be co-activated in immune cells by the TGF-β signaling pathway. Moreover, the Arg derivates spermidine and nitric oxide directly participate in modulating Trp metabolic pathway, by inducing or repressing IDO1 function, respectively. Given that Arg biosynthesis is controlled by the circadian clock (36), it might be very interesting to explore their reciprocal interaction in modulating the circadian immune response.

A prior study reported that *A. fumigatus* clearance from the lung varies with inoculation timing (27). In this study, lung fungal colonization was slightly increased after inoculation at ZT0 compared to ZT12, a result that seems to contrast with our finding of maximal colonization following inoculation at the beginning of the dark phase (ZT12). However, Chen et al. harvested lungs 14 h following infection, meaning that time of sampling were ZT14 and ZT2, opposite to the timing of infection. In our study the sampling time was 3 and 7 dpi, and samples were harvested at ZT3 when mice were infected at ZT3, and ZT12 when mice were infected at ZT12. Thus, the difference in the findings might suggests that both time of infection and time of organs collection should be considered as they might determine mild variations in colony number, especially during the first hours following infection.

The results of our study acquire greater relevance in specific clinical conditions, such as CF. In CF a hyper inflammatory state determines a non-resolving activation of the innate immune response, thus impairing microbial clearance and promoting a self-sustaining condition of progressive lung damage. Various pathogens, including *Aspergillus fumigatus*, colonizes CF airways and contributes to lung disease development and progression (37). The circadian clock control of immune functions is particularly evident in the respiratory system, where circadian rhythms actively control different aspects of lung pathophysiology (38, 39). Although partially defective in CF lungs, IDO1 maintains its circadian oscillation in basal conditions. Thus, CF mice develop a more severe pathogenic airway inflammation at the time of the day when IDO1 is less expressed. All in all, the identification of pathways that link the circadian rhythms to immune processes can pave the way for the identification of novel chronotherapeutic approach for the management of patients undergoing opportunistic infectious diseases, including fungal infections.

Although the introduction of a chronotherapeutic approach in the context of infectious disease is not straightforward, an antimicrobial circadian-based therapy could optimize our current approach and drive the therapy of infections according to the daily fluctuations of the host and, eventually, of the pathogen, thus maximizing its safety and efficacy. A crucial step in this direction will be the identification of pathways that link the circadian rhythms to infections in pre-clinical studies, followed by the development of reliable biomarkers that could confidently indicate the best timing for drug administration. Furthermore, it will be worth to study of the impact that currently available antimicrobial treatments might have on our circadian physiology, for a full transfer of knowledge to the bedside.

Lastly, it is worth to mention that the inflammatory response to fungi is also dependent on fungal morphotype (40, 41). Indeed, *A. fumigatus* resting versus germinating swollen conidia differently act on IDO1 activation and Treg generation, the first inducing and the second impairing IDO1 expression (42). Moreover, the Trp pathway in fungi plays a central role in the modulation of virulence, and IDO mutants produce more severe fungal infections in mice (43). Though very little is known regarding the presence of an intrinsic circadian clock in human fungal pathogens (44) and its potential relevance for the acquisition of virulence, the interaction between opportunistic fungal species and their hosts could have a profound influence in each other's behavioral and metabolic circadian activities, an almost unexplored field that could open up novel areas of investigation for therapy.

## Materials and methods

### *Aspergillus fumigatus* infection experiments

In the infection model, after induction of deep anesthesia with 3% isoflurane (Forane, Abbott) in oxygen, mice were instilled with 4 × 10^7^*A. fumigatus* (Af293) resting conidia per 20 μL of saline at the indicated ZTs (ZT3 and ZT12). Sacrifice was performed at 1, 3 and 7 dpi at ZT3 for mice infected at ZT3, at ZT12 for mice infected at ZT12. Quantification of fungal growth was performed as described (45). BAL fluid collection and morphometry were done as previously described (46). For differential BAL fluid cell counts, cytospin preparations were stained with May–Grünwald Giemsa reagents (Sigma-Aldrich). At least five fields/mouse were counted. Photographs were taken using a high-resolution BX51 microscope and images were captured using a DP71 camera (Olympus). For histology, paraffin-embedded sections were stained with periodic acid–Schiff (PAS) or Grocott-Gomori's methenamine silver stain.

### Flow cytometry

For flow cytometry, organs were processed as previously described (47). Data were analyzed on a FACS Fortessa flow cytometers (BD) with FlowJo software (Tree Star).

### Cytokine quantification

Mouse cytokines and chemokines were measured in lung homogenates using the Cytokine & Chemokine 26-Plex Mouse ProcartaPlex Panel 1 (Life Technologies), as indicated in the manufacturer's protocol.

### Cells and treatments

HEK293 cells were from ATCC (CRL-1573). WT HBE cells were provided by the primary cell culture service offered from the Italian Cystic Fibrosis Research Foundation and cultured as described (46). Total lung cells, alveolar macrophages and MEFs were obtained from C57BL/6 mice as described (46, 48). Zymosan from Saccharomyces cerevisiae (Sigma) was used at the final concentration of 50 µg/ml.

### QuantiGene plex gene expression assay

RNA preparation and quantification was performed as previously described (46). The sample input range was 1000 ng/well. Each sample includes RNAs from three mice per group, pooled before analysis. All QuantiGene probe sets, assay and data analysis were previously described (46). Centered-scaled heatmaps were evaluated on standardized expression values using R version 4.2.0 (49) and figures were produced using the package ggplot2 (50).

### qPCR

Mouse tissues and cells’ total RNA isolation, cDNA preparation and qPCR analysis were performed as described earlier (46). A complete list of the primers used in this study is provided in Table S1.

### HPLC analysis

Kyn and Trp concentrations in tissue homogenates were detected using a Perkin Elmer, series 200 HPLC instrument (MA, USA), as previously described (51).

### Western blot analysis

For Western blot analysis, lungs and ilea were harvested after treatment at the indicated time and processed as previously described (46).

### Statistics

GraphPad Prism software 6.01 was used for the analysis. Statistical significance was determined by 1 or 2-way ANOVA (Bonferroni's post-hoc test) for multiple comparisons and by the Student's *t-*test for single comparisons. Statistical significance was assumed for *P-*values of 0.05 or less.

## Supplementary Material

pgad036_Supplementary_Data

## Data Availability

All data that support the findings of this study are available in the manuscript and the Supplementary Materials.

## References

[1] Mohawk JA , Green CB, Takahashi JS. 2012. Central and peripheral circadian clocks in mammals. Annu Rev Neurosci. 35:445–462. 22483041 10.1146/annurev-neuro-060909-153128 PMC3710582

[2] Scheiermann C , Gibbs J, Ince L, Loudon A. 2018. Clocking in to immunity. Nat Rev Immunol. 18(7):423–437. 29662121 10.1038/s41577-018-0008-4

[3] Curtis AM , Bellet MM, Sassone-Corsi P, O'Neill LA. 2014. Circadian clock proteins and immunity. Immunity. 40(2):178–186. 24560196 10.1016/j.immuni.2014.02.002

[4] Nguyen KD , et al 2013. Circadian gene Bmal1 regulates diurnal oscillations of Ly6C(hi) inflammatory monocytes. Science. 341(6153):1483–1488. 23970558 10.1126/science.1240636 PMC3836670

[5] Bellet MM , et al 2013. Circadian clock regulates the host response to Salmonella. Proc Natl Acad Sci U S A. 110(24):9897–9902. 23716692 10.1073/pnas.1120636110 PMC3683799

[6] Gibbs J , et al 2014. An epithelial circadian clock controls pulmonary inflammation and glucocorticoid action. Nat Med. 20(8):919–926. 25064128 10.1038/nm.3599 PMC4268501

[7] Gagnidze K , et al 2016. Nuclear receptor REV-ERBalpha mediates circadian sensitivity to mortality in murine vesicular stomatitis virus-induced encephalitis. Proc Natl Acad Sci U S A. 113(20):5730–5735. 27143721 10.1073/pnas.1520489113 PMC4878505

[8] Edgar RS , et al 2016. Cell autonomous regulation of herpes and influenza virus infection by the circadian clock. Proc Natl Acad Sci U S A. 113(36):10085–10090. 27528682 10.1073/pnas.1601895113 PMC5018795

[9] Ehlers A , et al 2018. BMAL1 Links the circadian clock to viral airway pathology and asthma phenotypes. Mucosal Immunol. 11(1):97–111. 28401936 10.1038/mi.2017.24 PMC5638664

[10] Sengupta S , et al 2019. Circadian control of lung inflammation in influenza infection. Nat Commun. 10(1):4107. 31511530 10.1038/s41467-019-11400-9 PMC6739310

[11] Kiessling S , et al 2017. The circadian clock in immune cells controls the magnitude of leishmania parasite infection. Sci Rep. 7(1):10892. 28883509 10.1038/s41598-017-11297-8 PMC5589941

[12] Rijo-Ferreira F , et al 2018. Sleeping sickness is a circadian disorder. Nat Commun. 9(1):62. 29302035 10.1038/s41467-017-02484-2 PMC5754353

[13] Prior KF , et al 2018. Timing of host feeding drives rhythms in parasite replication. PLoS Pathog. 14(2):e1006900. 29481559 10.1371/journal.ppat.1006900 PMC5843352

[14] Costantini C , et al 2020. Tryptophan co-metabolism at the host-pathogen interface. Front Immunol. 11:67. 32082324 10.3389/fimmu.2020.00067 PMC7001157

[15] Munn DH , Mellor AL. 2013. Indoleamine 2,3 dioxygenase and metabolic control of immune responses. Trends Immunol. 34(3):137–143. 23103127 10.1016/j.it.2012.10.001 PMC3594632

[16] Pallotta MT , et al 2021. Indoleamine 2,3-dioxygenase 1 (IDO1): an up-to-date overview of an eclectic immunoregulatory enzyme. FEBS J. 289(20): 6099–6118. 34145969 10.1111/febs.16086 PMC9786828

[17] Platten M , Nollen EAA, Röhrig UF, Fallarino F, Opitz CA. 2019. Tryptophan metabolism as a common therapeutic target in cancer, neurodegeneration and beyond. Nat Rev Drug Discov. 18(5):379–401. 30760888 10.1038/s41573-019-0016-5

[18] Romani L . 2011. Immunity to fungal infections. Nat Rev Immunol. 11(4):275–288. 21394104 10.1038/nri2939

[19] Bell SC , et al 2020. The future of cystic fibrosis care: a global perspective. Lancet Respir Med. 8(1):65–124. 31570318 10.1016/S2213-2600(19)30337-6 PMC8862661

[20] Williams C , Ranjendran R, Ramage G. 2016. Pathogenesis of fungal infections in cystic fibrosis. Curr Fungal Infect Rep. 10(4):163–169. 28035247 10.1007/s12281-016-0268-z PMC5155017

[21] Lamia KA , Storch KF, Weitz CJ. 2008. Physiological significance of a peripheral tissue circadian clock. Proc Natl Acad Sci U S A. 105(39):15172–15177. 18779586 10.1073/pnas.0806717105 PMC2532700

[22] Storch KF , et al 2007. Intrinsic circadian clock of the mammalian retina: importance for retinal processing of visual information. Cell. 130(4):730–741. 17719549 10.1016/j.cell.2007.06.045 PMC2040024

[23] Chun SK , et al 2022. Disruption of the circadian clock drives Apc loss of heterozygosity to accelerate colorectal cancer. Sci Adv. 8(32):eabo2389. 35947664 10.1126/sciadv.abo2389 PMC9365282

[24] Fallarino F , et al 2006. The combined effects of tryptophan starvation and tryptophan catabolites down-regulate T cell receptor zeta-chain and induce a regulatory phenotype in naive T cells. J Immunol. 176(11):6752–6761. 16709834 10.4049/jimmunol.176.11.6752

[25] Keller M , et al 2009. A circadian clock in macrophages controls inflammatory immune responses. Proc Natl Acad Sci U S A. 106(50):21407–21412. 19955445 10.1073/pnas.0906361106 PMC2795539

[26] Gibbs JE , et al 2012. The nuclear receptor REV-ERBalpha mediates circadian regulation of innate immunity through selective regulation of inflammatory cytokines. Proc Natl Acad Sci U S A. 109(2):582–587. 22184247 10.1073/pnas.1106750109 PMC3258648

[27] Chen S , Fuller KK, Dunlap JC, Loros JJ. 2018. Circadian clearance of a fungal pathogen from the lung is not based on cell-intrinsic macrophage rhythms. J Biol Rhythms. 33(1):99–105. 29281921 10.1177/0748730417745178 PMC5858702

[28] Puccetti P , Grohmann U. 2007. IDO and regulatory T cells: a role for reverse signalling and non-canonical NF-kappaB activation. Nat Rev Immunol. 7(10):817–823. 17767193 10.1038/nri2163

[29] Iannitti RG , et al 2013. Th17/Treg imbalance in murine cystic fibrosis is linked to indoleamine 2,3-dioxygenase deficiency but corrected by kynurenines. Am J Respir Crit Care Med. 187(6):609–620. 23306541 10.1164/rccm.201207-1346OC

[30] Moravcová S , et al 2022. Circadian control of kynurenine pathway enzymes in the rat pineal gland, liver, and heart and tissue- and enzyme-specific responses to lipopolysaccharide. Arch Biochem Biophys. 722:109213. 35413276 10.1016/j.abb.2022.109213

[31] Petrus P , et al 2022. Tryptophan metabolism is a physiological integrator regulating circadian rhythms. Mol Metab. 64:101556. 35914650 10.1016/j.molmet.2022.101556 PMC9382333

[32] Mbongue JC , et al 2015. The role of indoleamine 2, 3-dioxygenase in immune suppression and autoimmunity. Vaccines (Basel). 3(3):703–729. 26378585 10.3390/vaccines3030703 PMC4586474

[33] Gargaro M , et al 2022. Indoleamine 2,3-dioxygenase 1 activation in mature cDC1 promotes tolerogenic education of inflammatory cDC2 via metabolic communication. Immunity. 55(6):1032–1050.e14. 35704993 10.1016/j.immuni.2022.05.013 PMC9220322

[34] Bellet MM , Zocchi L, Sassone-Corsi P. 2012. The RelB subunit of NFkappaB acts as a negative regulator of circadian gene expression. Cell Cycle. 11(17):3304–3311. 22894897 10.4161/cc.21669 PMC3467027

[35] Mondanelli G , Iacono A, Allegrucci M, Puccetti P, Grohmann U. 2019. Immunoregulatory interplay between arginine and tryptophan metabolism in health and disease. Front Immunol. 10:1565. 31354721 10.3389/fimmu.2019.01565 PMC6629926

[36] Lin R , et al 2017. CLOCK Acetylates ASS1 to drive circadian rhythm of ureagenesis. Mol Cell. 68(1):198–209.e6. 28985504 10.1016/j.molcel.2017.09.008

[37] King J , Brunel SF, Warris A. 2016. Aspergillus infections in cystic fibrosis. J Infect. 72:S50–S55. 27177733 10.1016/j.jinf.2016.04.022

[38] Nosal C , Ehlers A, Haspel JA. 2020. Why lungs keep time: circadian rhythms and lung immunity. Annu Rev Physiol. 82:391–412. 31561746 10.1146/annurev-physiol-021119-034602 PMC8818323

[39] Pariollaud M , et al 2018. Circadian clock component REV-ERBalpha controls homeostatic regulation of pulmonary inflammation. J Clin Invest. 128(6):2281–2296. 29533925 10.1172/JCI93910 PMC5983347

[40] Bozza S , et al 2005. A crucial role for tryptophan catabolism at the host/Candida albicans interface. J Immunol. 174(5):2910–2918. 15728502 10.4049/jimmunol.174.5.2910

[41] Levitz SM , Diamond RD. 1985. Mechanisms of resistance of Aspergillus fumigatus conidia to killing by neutrophils in vitro. J Infect Dis. 152(1):33–42. 2989387 10.1093/infdis/152.1.33

[42] Montagnoli C , et al 2006. Immunity and tolerance to Aspergillus involve functionally distinct regulatory T cells and tryptophan catabolism. J Immunol. 176(3):1712–1723. 16424201 10.4049/jimmunol.176.3.1712

[43] Zelante T , et al 2021. Aspergillus fumigatus tryptophan metabolic route differently affects host immunity. Cell Rep. 34(4):108673. 33503414 10.1016/j.celrep.2020.108673 PMC7844877

[44] Costantini C , et al 2020. Microbes in the era of circadian medicine. Front Cell Infect Microbiol. 10:30. 32117804 10.3389/fcimb.2020.00030 PMC7013081

[45] de Luca A , et al 2014. IL-1 receptor blockade restores autophagy and reduces inflammation in chronic granulomatous disease in mice and in humans. Proc Natl Acad Sci U S A. 111(9):3526–3531. 24550444 10.1073/pnas.1322831111 PMC3948220

[46] van de Veerdonk FL , et al 2022. Anakinra restores cellular proteostasis by coupling mitochondrial redox balance to autophagy. J Clin Invest. 132:e144983. 34847078 10.1172/JCI144983 PMC8759782

[47] Renga G , et al 2020. Thymosin alpha1 protects from CTLA-4 intestinal immunopathology. Life Sci Alliance. 3:e202000662. 10.26508/lsa.202000662 PMC7441522 32817121

[48] Iannitti RG , et al 2016. IL-1 receptor antagonist ameliorates inflammasome-dependent inflammation in murine and human cystic fibrosis. Nat Commun. 7:10791. 26972847 10.1038/ncomms10791 PMC4793079

[49] RCoreTeam (2014) R: A language and environment for statistical computing. Vienna, Austria: R Foundation for Statistical Computing.

[50] Wickham H . 2009. Ggplot2: elegant graphics for data analysis. New York: Springer.

[51] Albini E , et al 2018. Identification of a 2-propanol analogue modulating the non-enzymatic function of indoleamine 2,3-dioxygenase 1. Biochem Pharmacol. 158:286–297. 30391205 10.1016/j.bcp.2018.10.033

