# Supplementary material for: The circadian control of tryptophan metabolism regulates the host response to pulmonary fungal infections
Source: PNAS Nexus. 2023 Feb 3;2(3):pgad036. doi: 10.1093/pnasnexus/pgad036 (PMC9991457; doi:10.1093/pnasnexus/pgad036)
Supplement: pgad036_Supplementary_Data [file pgad036_supplementary_data.docx]

**
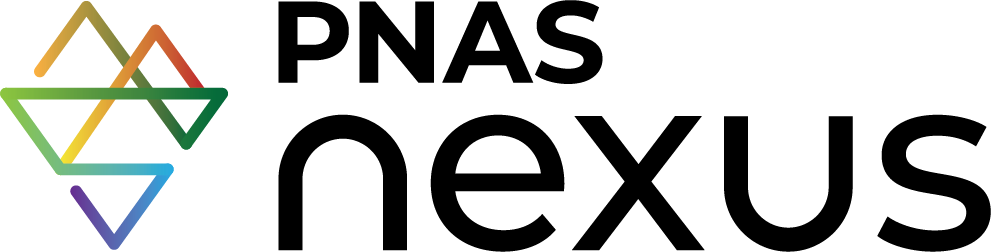
**

**Supplementary Information for**

The circadian control of tryptophan metabolism regulates the host response to pulmonary fungal infections

Claudia Stincardini^1^, Marilena Pariano^1^, Fiorella D’Onofrio^1^, Giorgia Renga^1^, Elena Orecchini^1^, Ciriana Orabona^1^, Emilia Nunzi^1^, Marco Gargaro^1^, Francesca Fallarino^1^, Sung Kook Chun^2^, Bridget M. Fortin^2^, Selma Masri^2^, Stefano Brancorsini^1^, Luigina Romani^1^, Claudio Costantini^1^ and Marina Maria Bellet^1^*

Marina Maria Bellet

Email: marinamaria.bellet@unipg.it

**This PDF file includes:**

Supplementary text (SI Materials and Methods)

Figures S1 to S9

Table S1

SI References

**SI Materials and Methods**

**Mice and infections.** 8- to 12-week-old male and female C57BL/6 WT mice were purchased from the Jackson Laboratory. Homozygous *Ido1^–/–^* raised on C57BL/6 background were bred at the animal facility of the University of Perugia, Perugia, Italy. CF mice homozygous for the Phe508del-Cftr allele, were obtained from B. Scholte (Erasmus Medical Center, Rotterdam, Netherlands) and generated as previously described (1). These mice were fed with a special food containing increased nutrients concentration (protein, lipids, fatty acids), protein quality (amino acid composition) and energy density to meet the potential higher requirements of these mice. Unless otherwise specified, animals were housed on a standard 12-hour light/dark paradigm and fed with standard chow ad libitum. The process of entrainment is the synchronization of circadian rhythm’s oscillation by a synchronizing agent, called zeitgeber. For circadian experiments in light/dark, light acts as external zeitgeber, and ZT is the standard of time, where the time of lights on usually defines ZT0 and lights off defines ZT12. For circadian experiments in constant darkness, also called free running conditions, the true endogenous circadian period can be revealed, and circadian time (CT) is the standard of time, where the activity onset of diurnal and nocturnal animals defines CT0 and CT12, respectively. For constant darkness experiments, animals were housed on a standard 12-hour light/dark paradigm for at least two weeks, and then transferred in constant darkness for 3 days before sacrifice. Mice tissues were harvested at the indicated ZT or CT. Intestinal-specific Bmal1 KO was achieved by crossing Villin-Cre mice and Bmal1 conditional mice that carry homozygous floxed alleles of exon 8 (2). These mice were purchased from The Jackson Laboratory. Whole gastrointestinal tracts were harvested at ZT3 and ZT12 from Bmal1 intestine-specific KO mice and their corresponding WT mice. All experiments were performed in accordance with the Italian Approved Animal Welfare authorization 360/2015-PR and Legislative Decree 26/2014 regarding the animal license approved by the Italian Ministry of Health (Rome) lasting for 5 years (2015–2020) and with the Institutional Animal Care and Use Committee (IACUC) guidelines at the University of California, Irvine. In the *C. albicans* infection model, *C. albicans* SC5314 cells were propagated on Sabouraud glucose agar at 37°C (Sigma-Aldrich, Milan, Italy). Gastrointestinal infection was performed by inoculating mice intragastrically with 1 x 10^8^ *C. albicans* cells in 200 mL saline using a 18G 4-cm-long plastic catheter, as previously described (3). Mice were sacrificed 3 and 10 days after infection. Fungal growth was expressed as colony-forming units (log10 CFU), obtained by serially diluting homogenates on Sabouraud agar plates incubated at 37 °C for 24 h.

**Flow cytometry.** Lungs were harvested, minced with scissors, and digested with Collagenase P (Sigma-Aldrich) and DNase in HBSS for 30 min at 37°C. For extracellular staining, cells were harvested by centrifugation at 1400 rpm for 5 minutes, followed by surface staining for 30 minutes at 4°C in 1x PBS + 0.5% BSA and 2 mM EDTA (MACS buffer) in presence of Fc blocking reagent (clone 2.4G). Subsequently, surface antigens were stained in MACS buffer for 30 minutes at 4°C. Extracellular staining was performed by using the following Abs: APC-CD3 (145-2C11; Biolegend), PE-DazzleTM-B220 (RA3-6B2; Biolegend), BV510-CD4 (RM4-5; BD Horizon), PerCP-Cy5.5-CD8 (53.6.7; BD Pharmingen), BV786-CD44 (IM7; BD Horizon). Cells were fixed and permeabilized for intracellular staining using the FoxP3/Transcription Buffer Set (eBioscience). Briefly, stained extracellular cells were fixed with 200 µl of Fixation/Permeabilization working solution for 30 minutes at 4°C, protected from the light. After twice washing in 200 µl of Permeabilization Buffer, the samples were incubated with PE-Foxp3 antibody (FJK-16s; eBioscience) diluted in Permeabilization buffer for 30 minutes at 4°C. After two washes with 200 µl of Permeabilization Buffer, cells were fixed with 1% (v/v) paraformaldehyde and analyzed on a FACS Fortessa flow cytometers (BD) with FlowJo software (Tree Star).

**Plasmids, transient transfection and luciferase Assay.** RAW264.7 cells were seeded in 24-well plates at a density of 7.5 × 10^4^ cells per well. A reporter constituted by the *Ido1* gene promoter (mIDOprom900-luc (4)), which contains the mouse *Ido1* promoter (900 bp) and 70 nucleotides of noncoding sequence in *Ido1* exon 1 upstream of the firefly luciferase coding sequence fused with the luciferase gene was transiently transfected in RAW 264.7, together with a plasmid expressing β-galactosidase for transfection control (LacZ pCMV). N-terminal myc-tagged plasmids myc-CLOCK-pSG5 and Flag-myc-BMAL1 pCS2+MT were previously described (5). Cells were transfected with Poly-jet (Tebubio), in accordance with the manufacturer’s instructions. Cell extracts were subjected to a luminometry-based luciferase assay, as described earlier (5), and luciferase activity was normalized by β-galactosidase activity.

**QuantiGene Plex gene expression assay.** Total RNA was extracted with TRIzol reagent (Thermo Fisher Scientific) and processed according to the instruction of the manufacturer. The total RNA was qualified and quantified by the Tecan Infinite 200 microplate reader (Tecan Group Ltd) following the instrument’s protocols. The RNA was diluted using nuclease-free water. The QuantiGene assays were performed as indicated in the manufacturer’s protocol. Plates were read using Magpix (Luminex). Data analysis was performed by normalizing the signals obtained for the genes of interest to the geometric mean of the reference gene signals (*Polr2a*, *Tfrc*, and *Hprt*).

**Quantitative PCR.** qPCR was performed using the CFX96 Touch Real-Time PCR detection system and iTaq Universal SYBR Green Supermix (Bio-Rad). Mouse tissues and cells were lysed, total RNA was isolated with TRIzol Reagent (Thermo Fisher Scientific), and cDNA was synthesized using the PrimeScript RT Reagent Kit with gDNA Eraser (Takara), according to the manufacturer’s instructions. Each data point was examined for integrity by analysis of the amplification plot. The thermal profile for SYBR Green RT-PCR was at 95°C for 3 minutes followed by 45 cycles of denaturation for 30 seconds at 95°C and an annealing/extension step of 30 seconds at 60°C. Amplification efficiencies were validated and normalized against *beta-actin*, *18S* or *Gadph*. Primers specific to the PCR templates of interest were designed using the free NCBI primer designing tool Primer-BLAST (<https://www.ncbi.nlm.nih.gov/tools/primer-blast/>). Primers were subsequently verified through BLASTN (standard nucleotide blast) (<https://blast.ncbi.nlm.nih.gov/Blast.cgi?PROGRAM=blastn&PAGE_TYPE=BlastSearch&LINK_LOC=blasthome>) and through a careful evaluation of qPCR efficiency and melting curve results at their first use. A complete list of the primers used in this study is provided in **Table S1**.

**HPLC analysis.** A Kinetex® C18 column (250×4.6 mm, 5 μm, 100 A; Phenomenex, USA), maintained at the temperature of 25 °C and pressure of 1800 PSI, was used. After deproteinization with perchloric acid 10%, tissue homogenates were injected and eluted by a mobile phase containing 10mM NaH_2_PO_4_ pH 3.0 (99%) and methanol (1%) (Sigma-Aldrich), with a flow rate of 1 ml/min. Kyn was detected at 360 nm and Trp was detected at 220 nm by an UV detector. The software TURBOCHROM 4 was used for evaluating the concentration of Kyn and Trp in samples by mean of a calibration curve. The detection limit of the analysis was 0.05 μM for Kyn and 0.5 μM for Trp. This method has been previously set to achieve the best resolution of peaks. Specifically, we identified the retention time of Kyn and Trp by using internal standards (Kyn and Trp) in tissue homogenates and by selecting the optimal column, mobile phase, temperature (25°C), and flow rate (1 ml/min).

**Western blot analysis.** Tissues were washed twice with cold PBS (Sigma-Aldrich), and lysed in RIPA buffer (Tris/HCl at pH 8.0, 50 mM, NaCl 150 mM, SDS 0.1%, sodium deoxycholate 1%, Triton X-100 1%), supplemented with protease inhibitor cocktail and PMSF (Sigma-Aldrich). Protein extracts were denatured by adding Laemmli buffer (Tris/HCl at pH 6.8, 200 mM, SDS 8%, bromophenol blue 0.4%, glycerol 40% and β-mercaptoethanol 5%) and boiled for 5 min at 95 °C. Proteins were separated by SDS-PAGE as described (6). Polyacrylamide gel (Bio-Rad) were transferred by electroblotting onto nitrocellulose membranes (Bio-Rad). Blots were blocked in dry fat-free milk 5% in PBS 1X for 1 h and then incubated with primary antibody overnight at 4 °C. Detection was achieved using horseradish-peroxidase-conjugated secondary antibody (Bio-Rad) and visualized with ECL (Bio-Rad). The following specific primary antibodies were used: IDO1 (cv152, as described in ref. (7)), p-STAT3, STAT3, p100/p52, p-STAT1, STAT1 (Cell Signaling Technology). Normalization was performed with β-actin, β-tubulin, or Gapdh antibodies (Sigma-Aldrich). Protein band intensities were quantified by densitometric analysis using ImageJ software.

**ChIP assay.** For lungs, 100 mg of lung tissue (100 mg) was processed following the dual cross-linking ChIP assay, as previously described (8). In detail, tissues were chopped into small pieces in 5 mL of PBS containing 1 mM MgCl_2_. Disuccinimidyl glutarate (DSG, Sigma-Aldrich) was added to a final concentration of 2 mM for cross-linking and the mix was incubated 45 min at room temperature. Formaldehyde was added to a final concentration of 1% (vol/vol) and samples were incubated for 15 min for dual cross-linking. The cross-linking was quenched by adding glycine to a final concentration of 0.1 M and the reaction was incubated for 10 min. Lung pieces were then centrifuged and pellets were homogenized in 2 mL of ice-cold PBS. After centrifugation, pellets were then resuspended in 1 mL of ice-cold cell lysis buffer [5 mM Hepes pH 8.0, 85 mM KCl, 0.5% Nonidet P-40, 1 mM PMSF, 1× protease inhibitor mixture (Sigma-Aldrich)] and incubated for 15 min on ice. Nuclei were precipitated by centrifugation (2,300 × g for 5 min), resuspended in 500 µL ice-cold RIPA buffer (50 mM Tris-HCl pH 8.0, 150 mM NaCl, 1% Nonidet P-40, 0.1% SDS, 0.5% sodium deoxycolate, 1 mM PMSF, 1× protease inhibitor mixture) and incubated on ice for 30 min. Sonication was performed to obtain DNA fragments 100–600 bp in length using Soniprep 150 (MSE Sanyo). Sheared DNA was subjected to IP using anti-BMAL1 antibody (Abcam) at 4°C overnight. Normal mouse IgG (Santa Cruz biotechnology) was used as the ChIP-negative control. The ChIP product was incubated with salmon sperm conditioned protein A Sepharose for 3 hours at 4°C to recover BMAL1-bound DNA. The resulting material was washed, collected in CHIP elution buffer containing 300 mM NaCl, 10 mM Tris pH 8.0, 0.5% SDS, and 5 mM EDTA pH 8.0, and then de-crosslinked, purified, and resuspended in ultrapure water. BMAL1 binding to chromatin was quantified using qPCR. The ChIP primers used for *Ido1* promoter are available upon request.


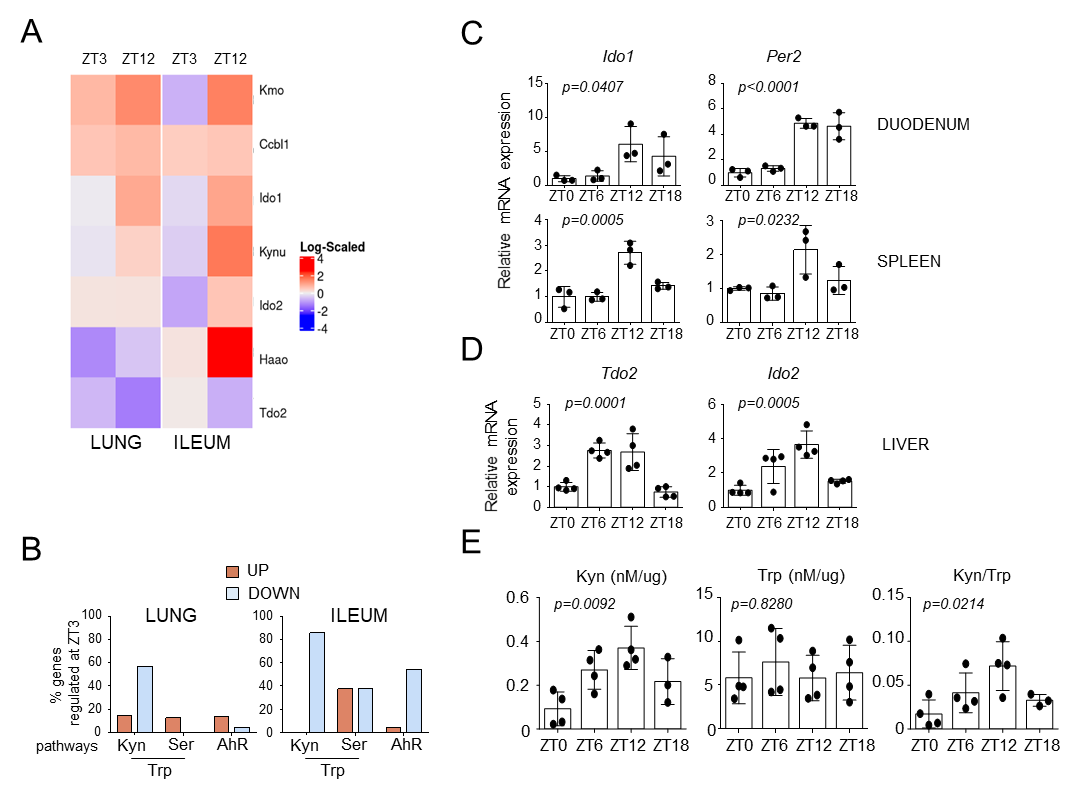


**Figure S1: Circadian regulation of the Trp metabolic pathway in different tissues.** **A)** Heat diagram of selected genes showing changes in gene expression detected by a custom QuantiGene plex gene expression assay in lungs and ilea of C57BL/6 mice collected at ZT3 and ZT12. Relative increase (red) or decrease (blue) of mRNA level is shown. **B)** Diagram showing the % of genes whose basal levels are up- or down-regulated (>20%) at day versus night in the Trp-Kyn pathway, the Trp-Ser pathway or the downstream AhR pathway. **C)** mRNA expression of *Ido1* and *Per2* in duodenum and spleen. **D)** mRNA expression of *Tdo2* and *Ido2* in liver. **E)** Levels of kynurenine (Kyn) and tryptophan (Trp), and Kyn/Trp ratio in liver homogenates. All values are the mean ± SD (n=3-4). One-way ANOVA, *p* values are shown.

**
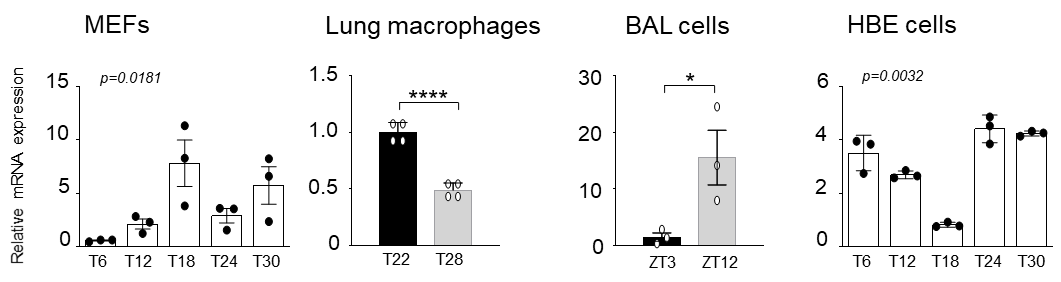
**

**Figure S2: Circadian *Ido1* oscillation in synchronized murine and human cells.** Circadian expression of *Ido1* in wild-type mouse embryonic fibroblasts (MEFs), lung macrophages, BAL cells and human bronchial epithelial (HBE) cells. Real-time PCR analysis was performed from RNAs prepared at the indicated ZTs or times after serum shock synchronization. Time 0 (unsynchronized cells) was set to 1. All values are the mean ± SEM (MEFs and BAL cells) or SD (lung macrophages and HBE cells) (n=3-4). One-way ANOVA or Student’s T test. Significant changes are shown, *p<0.05, ****p < 0.0001.


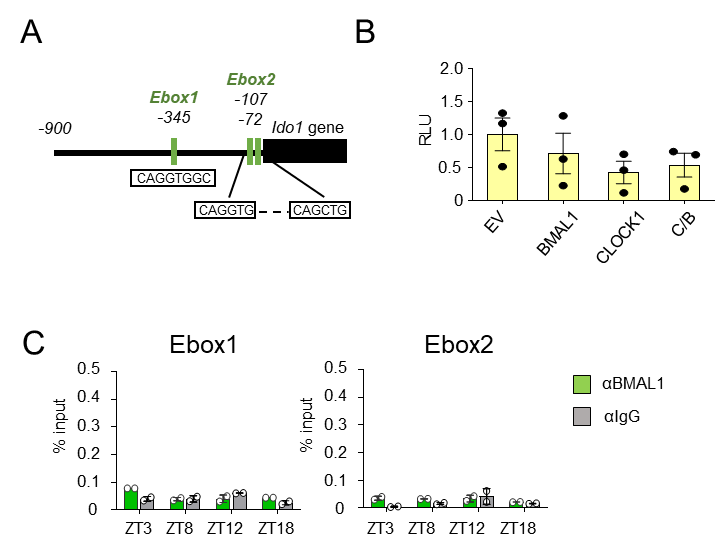


**Figure S3: *Ido1* promoter analysis. A)** Representative scheme of murine *Ido1* promoter. **B)** Vectors expressing CLOCK and BMAL1 (C/B) were cotransfected with a construct containing the m*Ido1*-luc promoter in RAW264.7 cells. The total DNA amount was kept constant by adding carrier plasmid DNA. After normalization for transfection efficiency using β-galactosidase activity, reporter gene activity was expressed as Relative Luciferase Units (RLU). Activity in control vector transfected cells was set to 1. All the values are the mean ± SD (n = 3). **C)** Chromatin immunoprecipitation from lungs of C57BL/6 mice collected at the indicated ZT, subjected to dual cross-link and immunoprecipitated with anti-BMAL1 and rabbit IgG. Primers for *Ido1* promoter region were used for quantitative PCR. All of the values are the mean ± SD (n = 2).


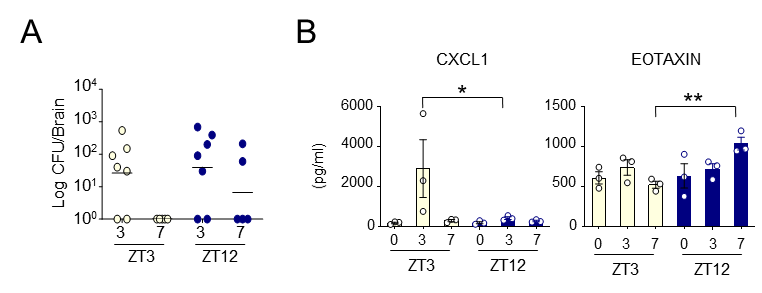


**Figure S4:** **Day-night susceptibility to *A. fumigatus* infection.** C57BL/6 wild-type mice infected with *A. fumigatus* and assessed at 3 and 7 days post-infection (dpi). **A)** CFU log10 count in the brain. Black bars indicate the geometric mean (n=5-7). Two-way ANOVA test, Bonferroni post-hoc test. No statistically significant changes were found. **B)** Mouse pro-inflammatory cytokines measured by multiplex immunoassay in lung homogenates. Results shown as mean ± SEM (n=3). p-values were generated by two-way ANOVA test, Bonferroni post-hoc test. Significant changes are shown. *p < 0.05, **p < 0.01.


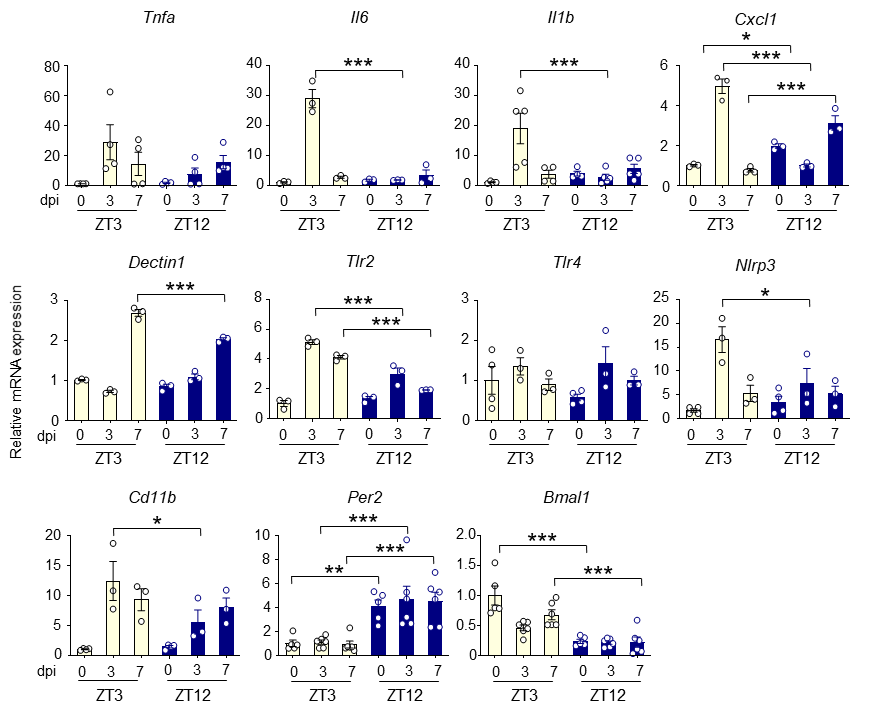


**Figure S5: Day-night cytokines expression during *A. fumigatus* infection.** mRNA expression of pro-inflammatory cytokines *Tnfa*, *Il6*, *Il1b*, chemokine *Cxcl1*, receptors *Dectin1*, *Tlr2*, *Tlr4,* *Nlrp3* and *Cd11b* and circadian genes *Per2* and *Bmal1*, evaluated by qPCR. All values are the mean ± SEM (n=3-6). Two-way ANOVA, Bonferroni post-hoc test. Significant changes are shown, *p < 0.05, **p < 0.01, ***p < 0.001.

**
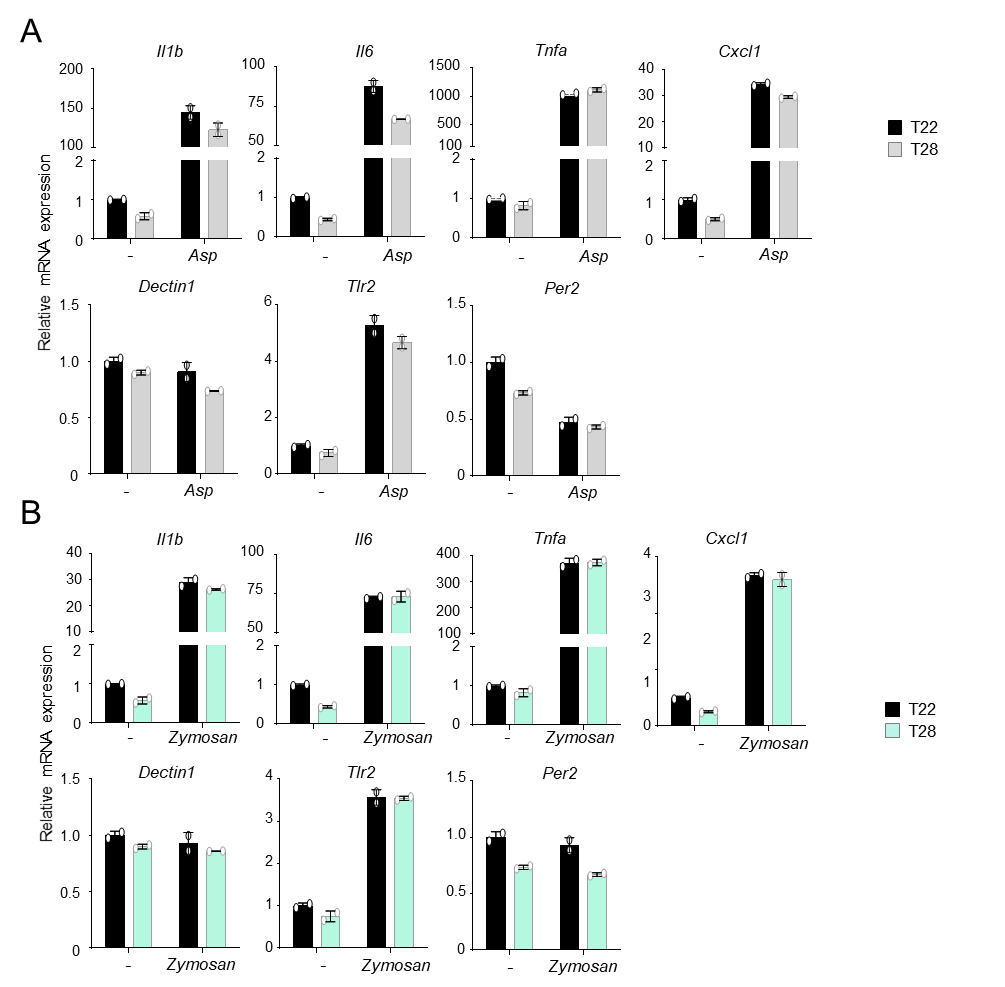
**

**Figure S6:** Cytokines production from synchronized lung macrophages. Circadian mRNA expression of pro-inflammatory cytokines and chemokines (*Il1b*, *Tnfa*, *Il6*, *Cxcl1*), fungal receptors (*Tlr2*, *Dectin1*), and circadian gene *Per2* in lung macrophages from C57BL/6 wild-type mice collected at T22 and T28 after serum shock synchronization and stimulated in vitro with *Aspergillus* or with Zymosan. All values are the mean ± SD (n=2).


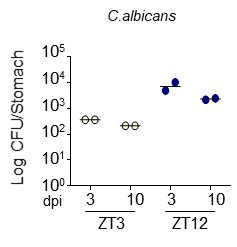


**Figure S7:** Day-night susceptibility to C. albicans infection. CFU log10 count in the stomach of C57BL/6 wild-type mice infected intragastrically with C. albicans at two ZT, and assessed at 3 and 10 day post-infection (dpi). Bars represent the geometric mean.


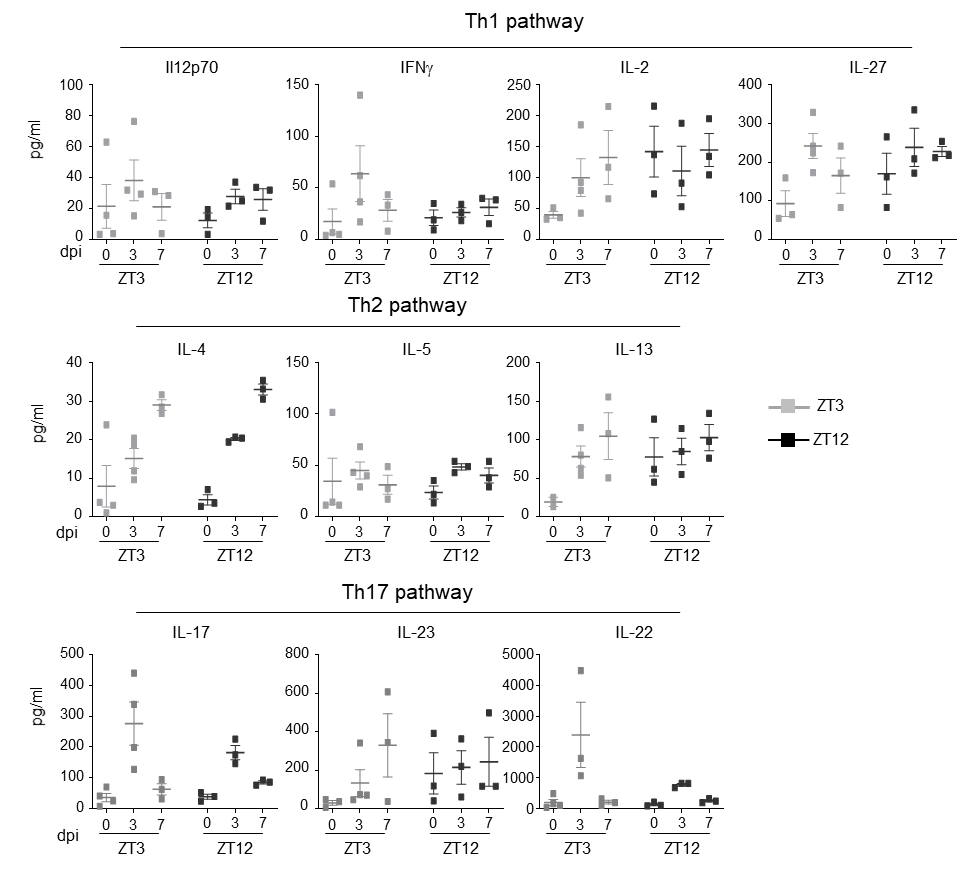


**Figure S8: Cytokines panel in infected lungs.** Mouse cytokines involved in Th1 (IL12p70, IFN-γ, IL-2, IL-27) **(A)**, Th2 (IL-4, IL-5 and IL-13) **(B)** and Th17 (IL-17, IL-23, IL-22) **(C)** pathway, measured by multiplex immunoassay in lung homogenates. Results shown as mean ± SEM (n=2-4). Two-way ANOVA, Bonferroni post-hoc test. No statistically significant changes were found.


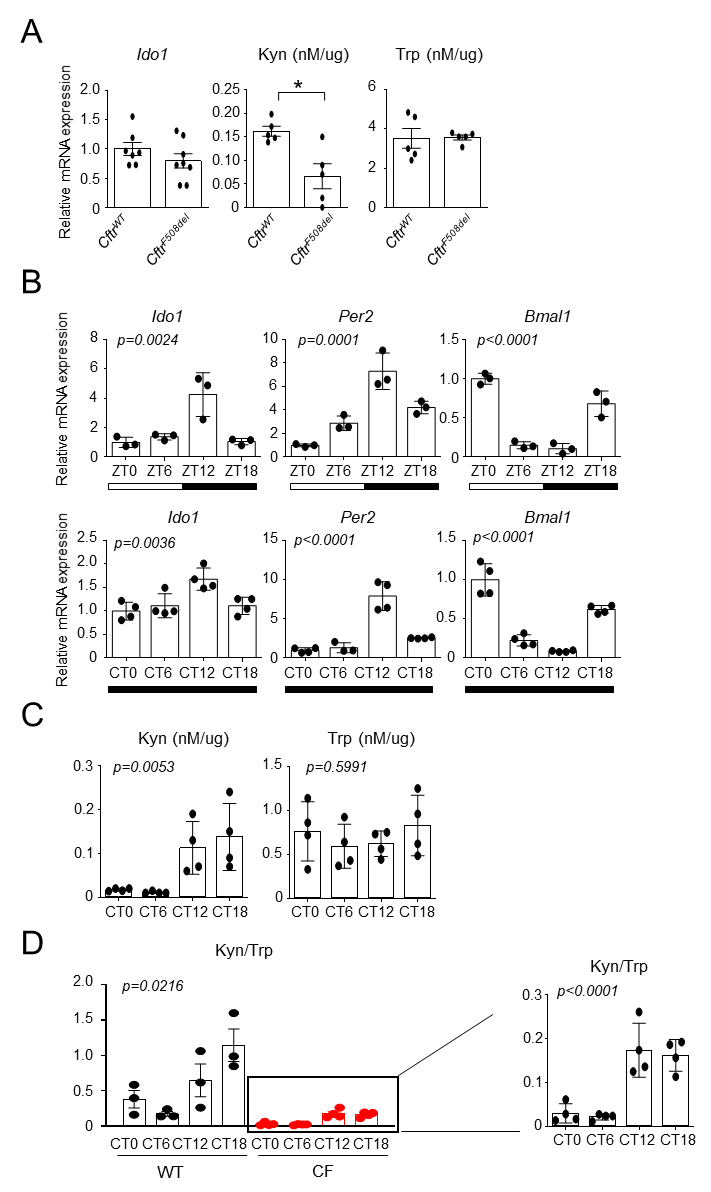


**Figure S9: *Ido1* circadian oscillation in CF mice. A)** Average *Ido1* gene expression, Kyn and Trp levels in lung homogenates from WT and CF mice taken at multiple ZTs. All values are the mean + SEM (n=5-7). Student’s T test, significant changes are shown. *p<0.05. **B)** Lungs from CF mice housed in 12h:12h light-dark or in constant darkness conditions were collected at four different ZT or CT, respectively. *Ido1*, *Per2* and *Bmal1* expression profiles were analyzed by quantitative PCR. Results shown as mean ± SD (n=3-4). One-way ANOVA, *p* values are shown. **C-D)** Levels of Kyn and Trp **(C)** and Kyn/Trp ratio **(D)** in lung homogenates from CF mice. All values are the mean ± SD (n=4). One-way ANOVA, *p* values are shown.

| **Gene** | **Primers sequence** | **References** |
| --- | --- | --- |
| *18S* | forward-CGGACACGGACAGGATTGACAG  reverse-ATCGCTCCACCAACTAAGAACGG | 6 |
| *beta-actin* | forward- AGCCATGTACGTAGCCATCC  reverse- CTCTCAGCTGTGGTGGTGAA | 3 |
| *Gapdh* | forward- TGTAGACCATGTAGTTGAGGTCA  reverse- AGGTCGGTGTGAACGGATTTG | 8 |
| *Bmal1* | forward- GCAGTGCCACTGACTACCAAGA  reverse- TCCTGGACATTGCATTGCAT | 9 |
| *Cxcl1* | forward- CCGCTCGCTTCTCTGTGC  reverse- CTCTGGATGTTCTTGAGGTGAATC | 3 |
| *Dectin1* | forward- CATCGTCTCACCGTATTAATGCAT  reverse- CCCAGAACCATGGCCCTT | - |
| *Foxp3* | forward- CCCAGGAAAGACAGCAACCTTTT  reverse- TTCTCACAACCAGGCCACTTG | 10 |
| *Haao* | forward- TTGGGGACAGCTATGAGACC  reverse- GCTCCCACACATACGAGGTT | 11 |
| *Ido1* | forward- CATGACATACGAGAACATGGAC  reverse- GACAGATATATGCGGAGAACG | 12 |
| *IDO1 (human)* | forward- TCACAGACCACAAGTCACAG  reverse- GCAAGACCTTACGGACATCT | 13 |
| *Ido2* | forward- CATGGCGCTGGCCGCTATCA  reverse- TTAAGGCCGGGCACTGCTGC | 12 |
| *Il1b* | forward- TGACGGACCCCAAAAGATGAAGG  reverse- CCACGGGAAAGACACAGGTAGC | 3 |
| *Il6* | forward- TTCCATCCAGTTGCCTTCTT  reverse- CAGAATTGCCATTGCACAAC | 5 |
| *Kmo* | forward- GCCTTGAAAGCCATTGGTC  reverse- GCACTGTGAGTACCCTTCC | - |
| *Kynu* | forward- ATATCATCACCCCGTCCAGA  reverse- CATCTGGTTCTCGCTTGTCA | - |
| *Nlrp3* | forward- ATGCTGCTTCGACATCTCCT  reverse- GTTTCTGGAGGTTGCAGAGC | 3 |
| *Per2* | forward- CGCCTAGAATCCCTCCTGAGA  reverse- CCACCGGCCTGTAGGATCT | 6 |
| *Tdo2* | forward- ATGAGTGGGTGCCCGTTTG  reverse- GGCTCTGTTTACACCAGTTTGAG | 12 |
| *Tgfb* | forward- ATATTTGGAGCCTGGACACA  reverse- CGTAGTAGACGATGGGCAGT | 3 |
| *Tlr2* | forward- CAGGTTCCAGTTTTCACCAC  reverse- CCATTGAGGGGTACAGTCGTC | - |
| *Tlr4* | forward- GCTTTCACCTCTGCCTTCAC  reverse- CGAGGCTTTTCCATCCAATA | - |
| *Tnfa* | forward- CGAGTGACAAGCCTGTAGCC  reverse- AAGAGAACCTGGGAGTAGACAAG | 3 |

**Table S1: qPCR primers used in this study listed in alphabetical order.**

**SI References**

1. van Doorninck JH*, et al.* (1995) A mouse model for the cystic fibrosis delta F508 mutation. *EMBO J* 14(18):4403-4411.
2. Storch KF*, et al.* (2007) Intrinsic circadian clock of the mammalian retina: importance for retinal processing of visual information. *Cell* 130(4):730-741.
3. Bellet MM*, et al.* (2021) Thymosin alpha 1 exerts beneficial extrapulmonary effects in cystic fibrosis. *Eur J Med Chem* 209:112921.
4. Pallotta MT*, et al.* (2014) Forced IDO1 expression in dendritic cells restores immunoregulatory signalling in autoimmune diabetes. *J Cell Mol Med* 18(10):2082-2091.
5. Bellet MM, Zocchi L, & Sassone-Corsi P (2012) The RelB subunit of NFkappaB acts as a negative regulator of circadian gene expression. *Cell Cycle* 11(17):3304-3311.
6. Bellet MM*, et al.* (2021) The Circadian Protein PER1 Modulates the Cellular Response to Anticancer Treatments. *Int J Mol Sci* 22(6).
7. Bessede A*, et al.* (2014) Aryl hydrocarbon receptor control of a disease tolerance defence pathway. *Nature* 511(7508):184-190.
8. Bellet MM*, et al.* (2013) Pharmacological modulation of circadian rhythms by synthetic activators of the deacetylase SIRT1. *Proc Natl Acad Sci U S A* 110(9):3333-3338.
9. Bellet MM*, et al.* (2013) Circadian clock regulates the host response to Salmonella. *Proc Natl Acad Sci U S A* 110(24):9897-902.
10. Renga G, *et al.* (2020) Thymosin α1 protects from CTLA-4 intestinal immunopathology. *Life Sci Alliance* 14;3(10):e202000662.
11. Renga G, *et al.*Optimizing therapeutic outcomes of immune checkpoint blockade by a microbial tryptophan metabolite. *J Immunother Cancer* 10(3):e003725.
12. van de Veerdonk FL*, et al.* (2022) Anakinra restores cellular proteostasis by coupling mitochondrial redox balance to autophagy. *J Clin Invest* 132(2).
13. Napolioni V, *et al.* (2019) Genetic Polymorphisms Affecting IDO1 or IDO2 activity differently associate with aspergillosis in humans. *Front Immunol* 10:890.
